# Dynamic connectivity and the effects of maturation in youth with attention deficit hyperactivity disorder

**DOI:** 10.1162/netn_a_00063

**Published:** 2018-12-01

**Authors:** Nina de Lacy, Vince D. Calhoun

**Affiliations:** Department of Psychiatry and Behavioral Science, University of Washington, Seattle, WA, USA; The Mind Research Network, Albuquerque, NM, USA; Department of Electrical and Computer Engineering, University of New Mexico, Albuquerque, NM, USA

**Keywords:** ADHD, Functional network connectivity, Dynamic connectivity, Cortical, Subcortical, ICA

## Abstract

The analysis of time-varying connectivity by using functional MRI has gained momentum given its ability to complement traditional static methods by capturing additional patterns of variation in human brain function. Attention deficit hyperactivity disorder (ADHD) is a complex, common developmental neuropsychiatric disorder associated with heterogeneous connectivity differences that are challenging to disambiguate. However, dynamic connectivity has not been examined in ADHD, and surprisingly few whole-brain analyses of static functional network connectivity (FNC) using independent component analysis (ICA) exist. We present the first analyses of time-varying connectivity and whole-brain FNC using ICA in ADHD, introducing a novel framework for comparing local and global dynamic connectivity in a 44-network model. We demonstrate that dynamic connectivity analysis captures robust motifs associated with group effects consequent on the diagnosis of ADHD, implicating increased global dynamic range, but reduced fluidity and range localized to the default mode network system. These differentiate ADHD from other major neuropsychiatric disorders of development. In contrast, static FNC based on a whole-brain ICA decomposition revealed solely age effects, without evidence of group differences. Our analysis advances current methods in time-varying connectivity analysis, providing a structured example of integrating static and dynamic connectivity analysis to further investigation into functional brain differences during development.

## Author Summary

Neuropsychiatric disorders represent a central field of inquiry in network neuroscience, akin to lesional analysis of complex brain system dynamics. The prevalence of these conditions increases from mid-childhood to peak in adolescence and regress thereafter, challenging our ability to disambiguate maturational and connectivity effects. ADHD is a paradigmatic example, and the most common neuropsychiatric disorder of development. Here we present the first whole-brain analysis of time-varying connectivity in ADHD. Building on leading-edge methods in dynamic connectivity, our novel approach analyses time-varying connectivity on both a global brain basis, and within local network systems. This framework demonstrates that analysis of time-varying connectivity offers additional ways to characterize group and maturational effects in ADHD that are extensible to other developmental neuropsychiatric disorders.

## INTRODUCTION

Historically, connectivity analysis of resting-state functional MRI (fMRI) data has largely proceeded by computing mean correlations between networks (or nodes) of interest across time courses. This provides an averaged snapshot of “static” functional network connectivity (sFNC), and superordinate approximation of underlying dynamic states (Ciric, Nomi, Uddin, & Satpute, 2017). Motivated by demonstrations that intrinsic connectivity fluctuates over the time series, methods have been in development since 2010 (Chang & Glover, 2010; Sakoglu et al., 2010) to delineate time-varying or *dynamic* functional network connectivity (dFNC) in resting-state fMRI, and capture brain network activity in richer detail. Concomitantly, secondary measures of dynamism have intuitive biological appeal as potential corollaries of cognitive control and flexibility (Nomi et al., 2017) to examine how the brain switches among prototype connectivity states over the time course of fMRI observations. In adults, state-dependent variability in intrinsic dFNC between fronto-parietal and default mode networks (DMN) is associated with reduced cognitive flexibility (Douw, Wakeman, Tanaka, Liu, & Stufflebeam, 2016), and dFNC variation permits automated identification of individual subjects and predicts fluid intelligence and executive function performance (Liu, Liao, Xia, & He, 2018). Initial investigations of the development of dynamism in youth, when cognitive control networks undergo considerable refinement entraining behavioral maturation, suggests increasing age is associated with increased variability in the time allocated to each state (Marusak et al., 2017) and greater switching fluidity in executive regions (Chai et al., 2017).

Although dynamic methods have yielded intriguing results in other developmental neuropsychiatric disorders (Damaraju et al., 2014; de Lacy, Doherty, King, Rachakonda, & Calhoun, 2017) revealing transient internetwork dysconnectivity that may be obscured by time-averaging, to date they have not been published in ADHD. This omission is remarkable given ADHD’s status as one of the most common neuropsychiatric developmental disorders and ample evidence of deficits in multiple executive functions (Mueller, Hong, Shepard, & Moore, 2017). Concomitantly, research in multiple fields suggests ADHD may be a convergent phenotype encompassing heterogeneous mechanisms (Gallo & Posner, 2016). Structural imaging studies have tended to support delayed maturation (Hoogman et al., 2017), with task-based region of interest studies suggesting lagging strengthening of fronto-parieto–striatal-cerebellar connections, with DMN, fronto-parietal, and ventral attention networks commonly implicated (Cortese et al., 2012; Hart, Radua, Mataix-Cols, & Rubia, 2012; Hart, Radua, Nakao, Mataix-Cols, & Rubia, 2013).

**Figure 1.**
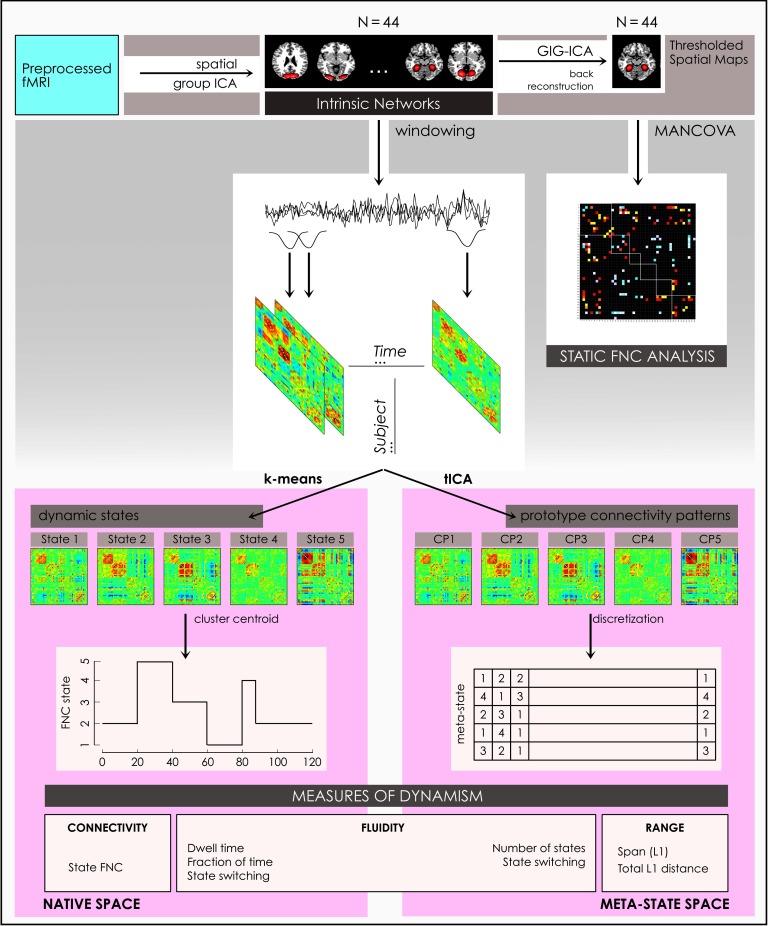
Computational Pipeline. A schematic of the computational pipeline illustrates steps in the preparation of the outcome measures of sFNC, dFNC, and dynamism. Preprocessed fMRI were submitted to spatial group ICA to extract 44 intrinsic networks, from which thresholded spatial maps are prepared via back reconstruction using the group information–guided ICA (GIG-ICA) algorithm. Pearson pairwise correlations averaged over the time courses for these spatial maps were analyzed using a multivariate analysis of covariance (MANCOVA) to form the basis for the static FNC analysis. In the dynamic pipelines, a sliding window approach to the intrinsic network (IN) time courses formed windowed FNC that were clustered using *k* means to estimate brain states, on which four measures of dynamism were computed in the native state space. Similarly, windowed FNC were clustered using the temporal ICA (tICA) algorithm to identify prototype connectivity patterns that were discretized to form the basis for four measures of dynamism computed in the meta-state space.

Studies in intrinsic FNC highlight key control networks and the DMN in ADHD, including relationships among DMN nodes. These networks play important roles in task-switching and “task-negative” states (Dosenbach et al., 2006, 2007). Although the relationship between control network roles and dFNC is largely undefined, this backdrop motivated our inquiry into time-varying connectivity in ADHD. We aimed to compare maturational effects, and those accruing from group membership, or diagnosis of ADHD, in a whole-brain analysis encompassing a full set of brain networks and dynamic measures. Because prior work has implicated disrupted connectivity among DMN nodes and the fronto-parietal network, we chose to estimate a higher order model. Higher order models enable the separation of right versus left fronto-parietal networks (Smith et al., 2009), and DMN subnetworks are increasingly understood as having differentiable functional emphases (Andrews-Hanna, Reidler, Sepulcre, Poulin, & Buckner, 2010). Furthermore, control networks, rich in association cortex, likely share multifunctional brain nodes to enable switching processes. Thus, techniques like ICA that allow individual nodes to participate in multiple networks support the discrimination of effects in control networks (Craddock, James, Holtzheimer, Hu, & Mayberg, 2012; Sepulcre, Sabuncu, & Johnson, 2012). To date, no published studies have examined dFNC in ADHD, or static FNC in a whole-brain ICA parcellation including subcortical regions, despite the prominent involvement of the latter in ADHD that is consistently demonstrated using task imaging. We aimed to address these gaps by analyzing sFNC and dFNC using a high-order ICA parcellation in a large age- and gender-matched subject sample drawn from the ADHD-200 repository, examining the effects of maturation and group (Figure 1).

Given evidence of altered DMN function in ADHD, and control network roles in functional configuration switching, we hypothesized that altered dynamism would be most prevalent within the DMN or in dFNC involving DMN subnetworks. We used ICA to estimate a 44-network model (Supporting Information Table S1, de Lacy & Calhoun, 2019) in 504 youth: 252 with ADHD and 252 with typical development (TD). We analyzed sFNC in a multivariate framework including group, age, IQ-level, gender, site, and residual motion effects, after determining comorbid diagnoses, medication usage, and handedness did not produce significant effects within the ADHD group. We then employed the *k*-means algorithm on windowed connectivity matrices (wFNC) to estimate four, five, and six brain states, identifying group differences in dFNC and measures of dynamic fluidity in this native state space. We removed the effects of gender, IQ-level, motion, and site by using the general linear model, successively including and then removing the effect of age. Finally, we identified four, five, and six prototype connectivity clusters by applying the temporal ICA (tICA) algorithm to wFNC. We discretized these to delineate a “meta-state” space and computed higher dimensional measures of dynamic fluidity and range, again with and without age effects and after removing variance attributable to IQ-level, gender, site, and motion. Five-state solutions are presented, and 4- and 6-state results reported. In addition, replication analyses were performed in a subsample of 444 subjects who were additionally matched for head motion in a 5-state solution.

We also present a novel increment to our prior work in dynamic methods by analyzing not only global, but also local, measures of whole-brain dynamism. We hypothesized that subjects with ADHD would exhibit decreased dynamism within the DMN system associated with group effects.

## RESULTS

### Group Differences in Static FNC Are Not Detectable in ADHD Versus TD Youth

Our analysis of static (averaged) FNC across all functional brain networks detected no significant difference in any pairwise correlation between ADHD and TD youth. Furthermore, there was no significant interaction of group with any other covariate including age and gender. These findings were replicated in the motion-matched sample. To examine the potential effects of comorbid diagnosis, history of prior or current medication usage, and handedness on sFNC, we performed an additional multivariate analysis in ADHD youth. By restricting the subject selection to ADHD youth, we took a conservative approach, reasoning any such effects would not be “diluted” by the presence of TD youth and including all subjects with a history of current or prior medication usage. We did not detect effects of comorbid diagnosis, medication usage, or handedness in sFNC, or interaction of these variables with group or age.

### Comparison of Maturational Effects in ADHD and TD Youth Shows Directional Differences in Control Networks and DMN Subnetworks

**Figure 2.**
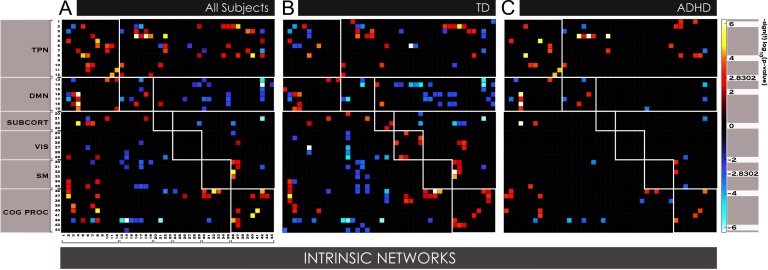
Maturational effects in static FNC among 44 INs in youth with ADHD vs. TD. Age effects in static connectivity in 252 subjects with TD and 252 with ADHD ages 7–17 was determined by computing Pearson pairwise correlations averaged across fMRI time courses among 44 INs obtained from ICA. In A, significant (α < 0.01 corrected for false discovery rate [FDR]) age effects are displayed for all 504 subjects, where B and C show significant (α < 0.05, corrected for FDR) age effects for TD and ADHD youth, respectively.

To explore the effects of maturation in sFNC, we analyzed age effects in all subjects together and in the ADHD and TD groups separately. Analysis of the effects of increasing age in sFNC in the entire subject group (Figure 2A) and in TD youth (Figure 2B) revealed several themes consistent with current understanding of connectivity changes in this developmental period. We observed age-driven strengthening in positive connectivity among networks within the same task-positive functional domains (network clades on the diagonal), except for the early maturing sensorimotor networks. Second, we found generally increasing anticorrelation between default mode subnetworks and noncontrol task-positive networks, indicating maturational segregation. Third, sFNC between task-positive control and DMNs showed a mixture of effects.Strengthening positive connectivity with increasing age was present involving dorsomedial and orbitofrontal (OFC) nodes with DMN, and between the ventral frontal portion of the ventral attention network, right fronto-parietal control network, and anterior cingulate portion of the cingulo-opercular network with precuneus subnetworks. In contrast, connectivity between the dorsal attention network and the DMN showed increasing anticorrelation with increasing age. Finally, among the task-positive control group itself, maturational segregation was also seen between the dorsal attention network and OFC, and between the dorsolateral prefrontal cortex and both OFC- and dorsomedial-anchored networks.

In youth with ADHD, we found increasing anticorrelation in fewer (sub)networks, which may be appreciated in Figure 2C where many more pairwise FNC cells are empty (black). Within the DMN system, youth with ADHD displayed increasing positive correlation between two DMN subnetworks anchored in the medial prefrontal cortex (intrinsic networks [INs] 16 and 18). Similarly, maturational effects in task-positive control networks and between task-positive and default mode subsystems differed in affected subjects. Task-positive control network relationships during typical development were characterized by increasing anticorrelation between subnetworks anchored in the prefrontal cortex: the dorsolateral prefrontal (IN6) with OFC (IN3), and dorsomedial prefrontal (IN4) cortices, and between the dorsal attention network (IN2) and OFC subnetwork (IN 3). This maturation segregation in prefrontal circuits was not observed in youth with ADHD. Instead, we detected increasing positive correlation with age between prefrontal circuits (IN6 and 7) and the anterior cingulate portion of the cingulo-opercular network (IN10), the ventral attention network (IN1) with right fronto-parietal control network (IN11), and dorsomedial prefrontal and temporo-limbic anchored circuits (IN 4 and 8). Maturation-driven strengthening of positive correlation between the dorsal attention network (IN2) and frontal cingulo-opercular network (IN 5) was common to subjects with TD and ADHD. There was no interaction of motion with these effects. In the motion-matched sample, most effects for each of the three analyses were replicated, as shown in Supporting Information Figure S1 (de Lacy & Calhoun, 2019). There were some differences, for the most part effects that did not appear in these smaller samples that were present in the larger samples without motion matching.

### Increased Global Dynamic Meta-State Span Was a Robust Predictor of ADHD Diagnosis Versus TD

We computed five measures of dynamic fluidity across the native (dwell time, fraction of time, state switching) and meta-state (L1 span, state switching) subspaces globally among all networks, and locally within network groups (Table 1). In the global analysis, where dynamism was examined among all networks on a whole-brain basis, L1 state span was significantly increased (α < 0.05, corrected for false discovery rate [FDR]) in ADHD > TD after age variance was removed. This finding was quite robust, being replicated in the 4-state and 6-state analyses, when window size was varied to 20TR or 30TR (where TR is the repetition time of the MRI acquisition sequence) across each of the 4-, 5-, and 6-state solutions, and in the motion-matched sample (Supporting Information Table S1, de Lacy & Calhoun, 2019). Other global meta-state measures of fluidity were significantly reduced (number of states achieved) or were reduced and narrowly missed significance (meta-state switching) in the 5-state analysis (Table 1) and in the motion-matched sample (Supporting Information Table S1), without replication in the 4-state and 6-state analysis.

**Table 1.**
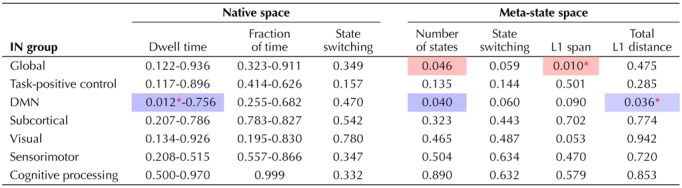
Global and local intrasystem dynamic fluidity and range measures in subjects with ADHD and TD.

*Note*. Statistical significance level (*p* value or α value) is displayed for differences in ADHD > TD for each measure of fluid dynamism in the 5-cluster analyses for global and within-system, network groupings. These values represent results after variance attributable to age was removed using the general linear model. Significant (*p* < 0.05 or α < 0.05, corrected for FDR) differences are highlighted in blue (ADHD < TD) and red (ADHD > TD). A red asterisk next to a value indicates a statistically significant solution was replicated to the same level of significance in 4-cluster and 6-cluster sensitivity analyses. Dwell time *p* values are shown in a range, indicating the lowest and highest *p* values in all five states. The DMN was the only system in which dwell time was significant, in only one state represented by the specific lower bound *p* value shown.

### Local Dynamic Fluidity Was Decreased Within the DMN System in ADHD

**Figure 3.**
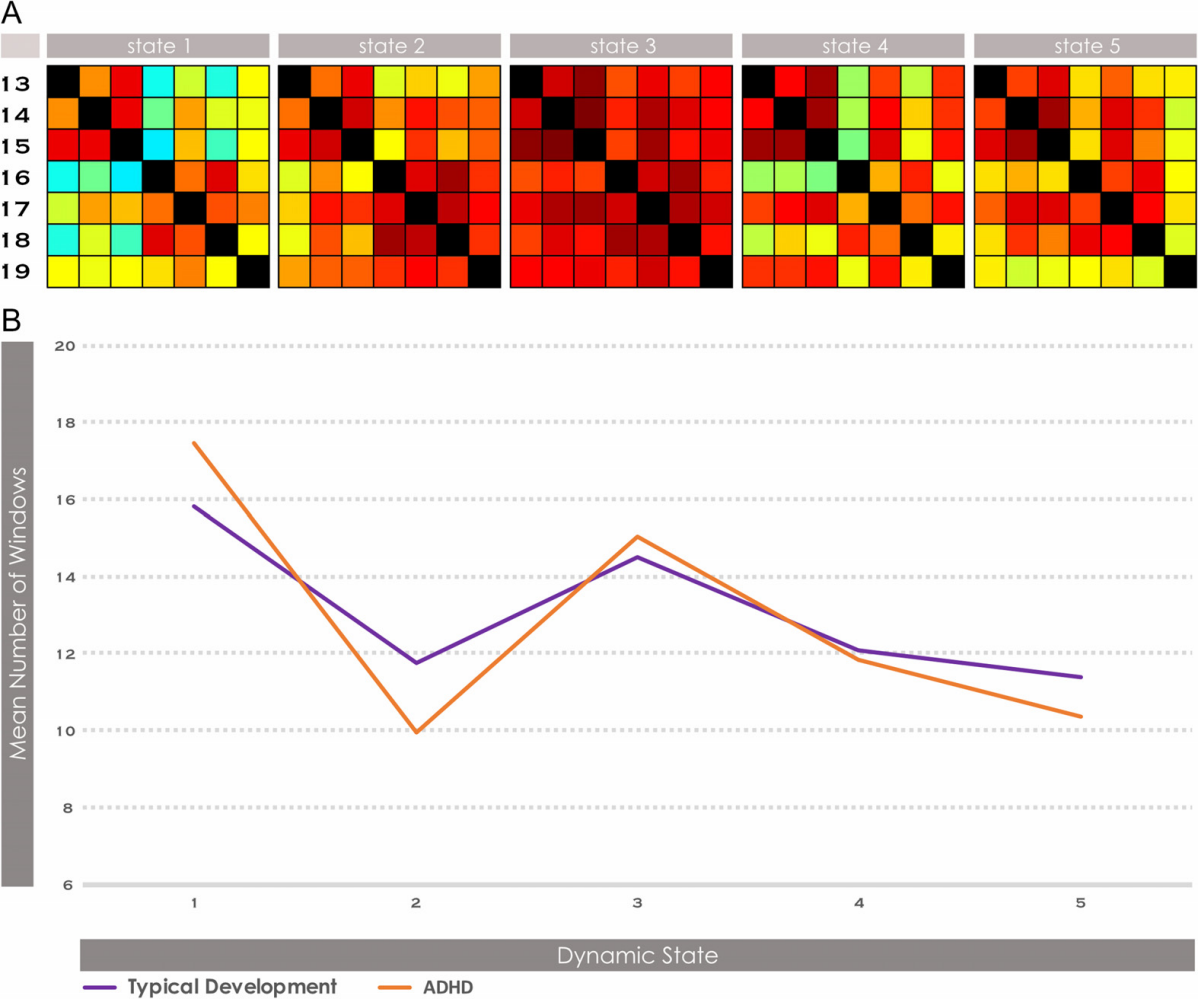
Brain states and local dwell time within the DMN system in ADHD vs. TD. Dynamic functional connectivity across five brain states was estimated with *k*-means clustering of windowed FNC from the ICA timecourses within the DMN system. The DMN system was represented in seven subnetworks, INs 13-19. See Supporting Information Table S1 (de Lacy & Calhoun, 2019) for further description of each subnetwork. The dwell time within each state was computed for each subject. Significant FNC differences between all combinations of DMN subnetworks, and significant differences in dwell time in ADHD > TD, were examined with two-sample *t* tests at a significant level of α < 0.05, corrected for FDR.

When we examined dynamism measures on a local basis (among subnetworks associated with the same neurocognitive function) in the native state space, we discovered significantly reduced dwell time in one state (State 2) in subjects with ADHD versus TD within the DMN system (Table 1 and Figure 3) that was characterized by high levels of connectivity among DMN INs. This finding was also present in the motion-matched sample (Supporting Information Table S1, de Lacy & Calhoun, 2019) and was replicated in the 6-state analysis. A directionally similar effect was present in the 4-state analysis, but missed significance at α = 0.128. In addition, we found significantly (α < 0.05, corrected for FDR) reduced L1 distance within the DMN system (Table 1), replicated in the 6-state analysis but not the 4-state analysis. Another measure of fluidity, meta-state switching, was significantly reduced in the 5-cluster analysis, very narrowly missing significance in the 4-state (α = 0.059) but not 6-state analysis. These latter two findings were not present in the motion-matched sample (Supporting Information Table S1, de Lacy & Calhoun, 2019).

Across all meta-state measures there was a generalized finding of reduced fluidity and range in ADHD versus TD within the DMN system, in every case either significant or narrowly missing significance. However, when subjects were motion-matched, no effect approached significance, although directionally there continued to be consistently reduced dynamism in ADHD. Differences in intrasystem dynamic fluidity never approached statistical significance in any other neurocognitive system (Table 1) in either the original or motion-matched subject samples.

We identified no significant (α < 0.05, corrected for FDR) differences in any measure of local fluidity or range between the DMN system and other network systems in ADHD (Table 2). However, some interesting general trends emerged that did not reach statistical significance in the original sample. We observed consistently reduced dynamism between the DMN and subcortical and sensorimotor systems, and consistently increased dynamism with task-positive control networks in ADHD versus TD across all meta-state measures. Similar trends were present in the 4-state and 6-state analyses. In the motion-matched sample, there was consistently increased dynamism between the DMN and all other neurocognitive systems in ADHD versus TD across all meta-state measures. In the case of task-positive control networks and DMN subnetworks, these achieved statistical significance in the number of states and state-switching measures (Supporting Information Table 2, de Lacy & Calhoun, 2019).

**Table 2.** Local DMN intersystem dynamic fluidity and range measures in subjects with ADHD and TD.

**IN groups**	**Native space**			**Meta-state space**			
	Dwell time	Fraction of time	State transitions	Number of states	State switching	L1 Span	Total L1 distance
DMN × task-positive control	0.337–0.867	0.829–0.986	0.769	0.602	0.602	0.467	0.831
DMN × sensorimotor	0.149–0.793	0.974	0.353	0.424	0.402	0.345	0.166
DMN × visual	0.281–0.912	0.988	0.521	0.459	0.466	0.712	0.666
DMN × subcortical	0.108–0.888	0.820–0.964	0.155	0.509	0.550	0.079	0.311
DMN × cognitive processing	0.186–0.852	0.333	0.910	0.618	0.664	0.510	0.912

*Note*. Statistical significance level (*p* value or α value) is displayed for differences in ADHD > TD for each measure of fluid dynamism in the 5-cluster analyses for cross-system grouping between the DMN group and every other network group. All values represent two-sample *t* tests performed at *p* < 0.05 or α < 0.05, corrected for FDR. These values represent results after variance attributable to age was removed using the general linear model. All results replicated to the same level of significance in the 4-state and 6-state analyses with the exception of a significant difference in dwell time between the DMN and visual system in State 2 in the 4-state analysis. Dwell time and fraction of time values are shown in a range, indicating the lowest and highest bounds for each of the five states.

### Transient DMN-Striato-Thalamic Hyperconnectivity Associated with a Diagnosis of ADHD Was Revealed by Local dFNC Analysis

**Figure 4.**
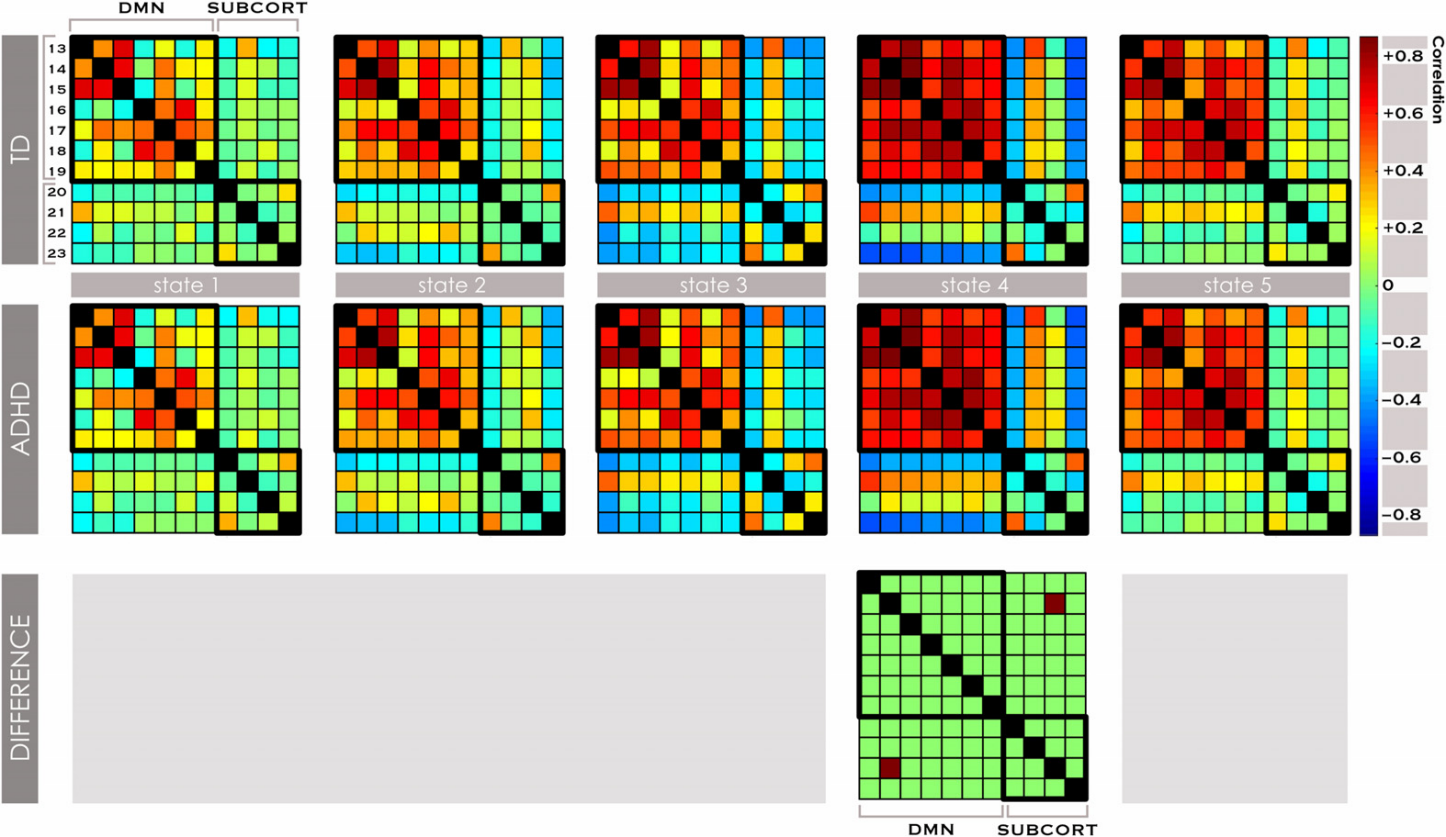
Transient hyperconnectivity between the posterior DMN and striato-thalamic network in dFNC states. Dynamic functional connectivity across five brain states was estimated with *k*-means clustering of windowed FNC from the ICA timecourses in global and local intrinsic connectivity systems. In the global analysis, differences in FNC between subjects with ADHD and TD were examined across all networks considered simultaneously. In local analyses, differences were computed within subnetworks of each functional system and between the DMN system and every other system. Significant FNC differences between any combination of intrinsic network systems were examined with two-sample *t* tests at a significant level of α < 0.05, corrected for FDR.

We identified a single, robust finding in analysis of FNC within five brain states that replicated in the 4-state and 6-state analysis. In local dFNC between the DMN and subcortical systems, subjects with ADHD displayed significant hyperconnectivity between a posterior cingulate-anchored subnetwork of the DMN (IN14), and the basal ganglia-thalamic network (IN30). This was a transient phenomenon that appeared in one brain state (Figure 4). Of note, this was not detectable in the sFNC whole-brain analysis of either group or maturational effects, further suggesting it is a local dynamic feature attributable to the effect of ADHD diagnosis. In each of the 4-, 5-, and 6-state analyses, this functional state was characterized by strong correlations among DMN subnetworks, suggesting it corresponds to DMN activation. Additionally, while anticorrelation between the DMN and striatal/cerebellar subnetworks is common among dFNC states, it was significantly stronger in this state. In the motion-matched sample of 444 youth, this effect also appeared, similarly in a state where there were strong correlations among DMN subnetworks and anticorrelation between DMN and subcortical subnetworks. Interestingly, in the motion-matched subsample there were additional significant effects extending across all states, the majority also being hyperconnectivity, with 60% of all effects involving the same basal ganglia-thalamic network. These may be inspected in Supporting Information Figure S2 (de Lacy & Calhoun, 2019).

We also identified significant group differences in localized dynamic connectivity in the native state space. Specifically, we found evidence of hyperconnectivity between two cognitive processing networks when local dFNC was analyzed within the cognitive processing network group: IN38, a network anchored in retrosplenial/parahippocampal cortex associated with memory and perceptual areas, and IN4, associated with expressive speech. This finding was not replicated in the 4-state or 6-state analysis, or in the motion-matched sample. Other than these findings, there were no significant differences in dFNC in any other of the global or local measures of dynamic connectivity. In the motion-matched sample, we also detected significant differences in dFNC between DMN subnetworks and visual networks, which may be viewed in Supporting Information Figure S3 (de Lacy & Calhoun, 2019).

## DISCUSSION

Notwithstanding its interest to the neuroimaging research community, surprisingly few studies have been published in youth with ADHD utilizing ICA to assess intrinsic connectivity between large-scale networks. In intrinsic sFNC, a combination of group and maturational effects have been identified. A trio of studies in the ADHD-200 data by the same group represents the largest body of work to date. These use a grid-based 907-seed parcellation restricted to cortex, in 133 youth with ADHD and 288 TD subjects (the latter skewing older and female), performing scrubbing in up to 60% of frames to examine sFNC. Collectively, they identified group effects in hypoconnectivity of the DMN with fronto-parietal control, ventral attention and visual networks, and within-DMN. In a separate study, maturational lag was identified in these same network pairs (C. Sripada et al., 2014; Sripada, Kessler, & Angstadt, 2014). Using joint-ICA, hyperconnectivity was seen in DMN, again with ventral attention and fronto-parietal control, and also the dorsal attention network (Kessler, Angstadt, Welsh, & Sripada, 2014). A separate group performed one of the few extant analyses using ICA, in 40 age- and gender-matched children in 12 networks, finding altered maturational connectivity maturation between salience and sensorimotor networks, and anterior-posterior nodes of the DMN (Choi, Jeong, Lee, & Go, 2013). In addition, a specific contribution of connectivity-driven studies focused on internetwork relationships has been the generation of the “Default mode network interference hypothesis” in ADHD. This proposes that attenuated DMN deactivation in task-demanding states and reduced segregation between the DMN and task-positive control networks may be associated with deficits in the appropriate functioning of the latter (Castellanos & Aoki, 2016; Castellanos & Proal, 2012; Sonuga-Barke & Castellanos, 2007). It has been further suggested that this results from abnormally reduced suppression of medial prefrontal DMN nodes (Fassbender et al., 2009).

Using an ICA-based functional parcellation, we could not attribute sFNC differences to group (ADHD) effects as detected by (Sripada et al., 2014), even though we used the same underlying dataset, in either our original or motion-matched sample. This latter discrepancy may be due to methodologic and subject-selection differences. We constructed an approximately age- and gender-matched subject sample, since older age and female gender are both protective factors, and may therefore artificially magnify ADHD > TD differences. In addition, we used ICA to obtain a data-driven functional parcellation rather than a post hoc assignment of edges to spatial maps, addressing motion via regression of motion parameters, selective despiking, removal of motion artifact via the ICA decomposition, and controlling for motion in the statistical analysis, rather than scrubbing.

Our analysis of maturational effects in sFNC in youth was consistent with the established literature, with increasing positive connectivity among task-positive control networks and between these networks and other task-specific networks such as visual or sensorimotor networks, and increasing anticorrelation between DMNs and task-specific networks. We also performed an exploratory comparative analysis by splitting the subject group into TD and ADHD groups with equal power that were approximately balanced for age and gender. These analyses showed directional differences. In youth with ADHD, the maturational segregation seen in TD among certain posterior subnetworks of the DMN was not present, congruent with recent work (Mills et al., 2017), but instead age-related strengthening between medial prefrontal cortical subnetworks was identified. In TD, there was a general theme of strengthened coupling between the DMN and large-scale circuits anchored in dorsomedial and orbitofrontal cortex. In ADHD, the former was also present, but orbitofrontal maturational strengthening with DMN was not. The OFC is linked to reward-based decision-making and impulsivity, both consistent parts of the ADHD phenotype (Mueller et al., 2017). Similarly, in ADHD youth we found maturational strengthening of positive couplings between precuneus and the anterior cingulate node of the cingulo-opercular network as in TD youth, but the increasing maturational correlation seen in TD youth between the ventral frontal portion of the ventral attention network and the right fronto-parietal control network was not present in ADHD. These latter networks have been consistently linked with ADHD and these findings may link to recent research demonstrating a differentiated pathway of precuneus functional development from the rest of the DMN (Yang et al., 2014). Finally, segregation of the dorsal attention network with dorsolateral prefrontal cortex was present in TD youth, but not those with ADHD. As we note previously, it has been suggested that higher motion may be a trait difference in ADHD, but this is not a settled issue and therefore motivated our replication analyses in a motion-matched subsample. This replication analyses in sFNC in a motion-matched subsample reassuringly detected very similar effects with minor selected differences which may be due to motion differences, or to reduced power in this smaller sample. Overall, our high-order model provides additional detail on connectivity maturation among subnetworks of the larger networks commonly considered in lower order models, showing directionally divergent patterns of sFNC maturation, but no identifiable group effects, in youth with ADHD and TD.

In contrast, our analysis of time-varying intrinsic connectivity based on ICA-derived networks demonstrated group differences associated with ADHD diagnosis. We identified transient hyperconnectivity between the basal ganglia-thalamic network (IN30) and a subnetwork of the DMN anchored in the posterior cingulate (IN14). This phenomenon was robust to variance in analytic parameters and provides the first conceptual link between decades of structural and task-based findings in ADHD implicating the striatum, and dynamic connectivity. Furthermore, in the motion-matched subsample even more dFNC effects were detected, the preponderance involving the same basal ganglia-thalamic network. Of note, the effect in the original sample occurred in a distinctive brain state where maximal anticorrelation obtained between the DMN and subcortical system generally, and strong positive connections were observed within the DMN system. In the motion-matched subsample, the largest number of dFNC differences were also in this state. An intriguing conceptual parallel is present in our earlier work in schizophrenia, where we identified that thalamico-cortical dFNC differences with TD existed in states with the strongest cortical-subcortical anticorrelations (Damaraju et al., 2014). Similarly, in autism we found dFNC differences occurred in states exhibiting greater anticorrelation between the DMN and other network systems (de Lacy et al., 2017). Although the specific networks involved differ among these conditions, these findings suggest that dynamic configuration states where the DMN is more anticorrelated with task-positive networks may represent increased vulnerability to transient dysconnectivity phenomena associated with neuropsychiatric conditions.

Our analysis of global measures of dynamism provided further group-specific effects. In particular, we identified a significantly increased global meta-state span in ADHD > TD. This finding suggests that subjects with ADHD traverse a larger whole-brain state space to instantiate basal connectivity patterns, perhaps representing a more inefficient dynamic “search process.” We believe the higher dimensional meta-state measures are generally less susceptible to distortion than those native state spaces, and since this measure was quite robust to variations of our analytic parameters and appeared in the motion-matched sample. It differentiates ADHD from at least one other neuropsychiatric condition (Miller et al., 2016), and may offer a connectivity motif with specificity for ADHD. In this study, we introduced a novel increment of our prior work in dynamic analysis by introducing local measures of dynamism within network systems. These were developed to test for dynamic effects that localized to the DMN system, and they produced three significant results. In every case, dynamism was significantly reduced in ADHD < TD within the DMN, extending to both fluidity and range, and associated with ADHD diagnosis. Although only the dwell time measure replicated in the motion-matched sample, we did similarly detect reduced dynamism in all local DMN measures. This suggests a specific phenomenon exists of reduced dynamism within the DMN system, among subnetworks of the DMN, in youth with ADHD. In contrast, we detected significantly increased local dynamism across a number of measures in the task-positive control networks in our motion-matched sample, directionally similar to effects that narrowly missed significance in the original sample. These contrasted with other neurocognitive network types where there were no such trends. Overall, our results suggest group differences in connectivity in ADHD are characterized by themes of increased dynamism in the task-positive control network system, and decreased dynamism within the DMN system.

## LIMITATIONS

Although our high-order model provides visibility into subnetwork-level connectivity such as relationships among DMN nodes, correction for multiple comparisons is concomitantly increased. Thus, our results may be overly conservative or different effects might be identified in a lower order model. More specifically, while the global dynamic connectivity measures correct for at least the same number of comparisons as the sFNC analysis, the local system analyses and secondary measures of dynamism undergo less correction, because fewer INs are compared. Although we selected model parameters for the dynamic analyses based on external criteria and prior work, and performed sensitivity analyses on many portions of the study, we did not perform exhaustive sensitivity analysis. For example, while cluster number was varied between 4 and 6 for all measures, we only varied window size for the global analysis. Thus, it is possible that further variance in model parameters might influence results. Clustering and dynamic connectivity analysis are related and continuously evolving fields, and we expect that future work will continue to refine available methods. We sorted ICA components into gray-matter INs and artifactual components by using a conventional semimanual process, which is a potential limitation: fully automated sorting and labeling of ICA components remains an area of active ongoing research (Calhoun & de Lacy, 2017). Our study overall may be limited by power considerations, for example, in considering effects of comorbidity or medication usage. In addition, the effects of head motion are an ongoing concern in ADHD and imaging youth more generally. In this study, which focused on dynamic connectivity, we have approached the issue of motion by using multiple methods: excluding high-motion subjects, regressing realignment parameters, performing despiking, and ICA. We retained a significant difference between groups, given concern this might be a trait difference, although an alternative approach would be to match subjects based on head motion. We also covaried for DVARS to check for the effects of residual motion. However, other approaches are available such as scrubbing, and methodologic differences might influence results. Finally, this study was performed in cross-sectional data, acting only as a proxy for maturational effects. Future studies in larger and longitudinal subject samples such as the ABCD project will offer exciting opportunities to extend and refine dynamic connectivity approaches in ADHD.

## CONCLUSION

Taken together, our findings suggest that localized, reduced dynamism among multiple subnetworks of the DMN and increased dynamism among task-positive control circuits in ADHD are associated with transient hyperconnectivity between the DMN subnetworks and striato-thalamic circuits, and increased whole-brain global dynamic fluidity. This aberrant DMN and control network dynamism offer connectivity markers associated with a diagnosis of ADHD. Our analysis based on networks identified using ICA indicates a central role for the DMN in ADHD, but does not support findings of disrupted FNC associable with group effects outside transient time-varying FNC disruption in striato-thalamic and other subcortical circuits, and perhaps visual INs. Overall, our work suggests that dynamic connectivity analysis holds promise to differentiate ADHD from other major developmental neuropsychiatric conditions with which it is frequently comorbid such as autism and schizophrenia, and identify mechanisms contributing to the general ADHD phenotype. Further research will inform the specificity of these findings.

## METHODS

### Functional MRI Processing

Unprocessed resting-state fMRI data for subjects with ADHD and TD ages 7.0–17.9 were obtained from the ADHD-200 repository and matched for age and gender. The study was declared exempt from human subjects research considerations by the University of Washington Institutional Review Board. The first five time points were discarded from the beginning of each scan to account for possible MRI equilibration effects. Scans were slice-time corrected to the middle scan volume, realigned to the first image in the series, and coregistered and normalized to the functional template by using standard algorithms in SPM12. After processing, data were submitted to quality control to assess the quality of the normalization and degree of subject motion by computing (1) spatial regression between each normalized functional image and a group mask constructed from all subjects and (2) root-mean-square difference of volume N to volume N + 1, also known as DVARS (Christodoulou et al., 2013; Power et al., 2014). Subjects with <95% correspondence between their normalized image and the mask, and subjects with motion >2 standard deviations from the mean DVARS were eliminated from consideration.

### Construction of Subject Sample

Our goal was to create a subject sample that was approximately representative of the youth population with ADHD in terms of age distribution, handedness, comorbidities, gender, and head motion while preserving statistical power given the effect size in resting-state imaging. Subjects passing imaging quality control criteria were selected to create an approximately age- and gender-matched sample of 252 youth with ADHD and 252 TD youth, ages 7.0–17.9, with demographic characteristics summarized in Table 3.

**Table 3.** Demographic characteristics of subjects.

**Age group**	**Typically developing**		**ADHD**	
	Number	% Male	Number	% Male
7–8.99	38	52.6	40	65.0
9–10.99	72	62.5	73	71.2
11–12.99	65	78.5	60	80.0
13.0–14.99	51	94.1	54	94.4
15.0–17.99	26	80.8	25	80.0
Total	252	73.4	252	78.1

Age and IQ for this sample were normally distributed, with skew and kurtosis between −3.5 and +3.5. Subjects had full-scale IQ (FSIQ) ranging from 73 to 153 with a mean of 110. There was no significant difference in FSIQ between groups. Our sample contains 3:1 male-to-female ratio and is weighted toward children over 11 years old, directionally similar to the distribution of clinical incidence of ADHD in the population. Within the younger age bands, we included a slightly greater proportion of females given our desire to include more younger subjects and the relative paucity of male controls available at this age. Our 504 subjects came from the Peking, KKI, NYU, and Pittsburgh sites in the ADHD-200 repository. Individual variables (regressors) were constructed for each site. A list of included subjects with their ID codes, corresponding to that given in the ADHD-200 repository key, may be inspected in Supporting Information Table S3 (de Lacy & Calhoun, 2019).

The clinically diagnosed ADHD population is enriched for left-handedness, although its significance is disputed (Ghanizadeh, 2013). We retained this characteristic, and there was a significant difference in handedness scores between groups (*p* = 0.003) with more left-handedness in the ADHD group. Similarly, in the ADHD group, 96 subjects (38%) reported past or present comorbidity with specific learning disorders, oppositional defiant disorder, depression, anxiety disorders, and Tourette’s syndrome, approximately equivalent to the general population with ADHD. No subjects reported autism, schizophrenia or psychotic symptoms. Instruments used to make diagnoses of ADHD and comorbidities varied across site—a limitation—and these may be inspected at http://fcon_1000.projects.nitrc.org/indi/adhd200/. Fifty-nine subjects in our sample with ADHD were reported to not be naïve to psychoactive medications. Of these, three subjects in the Pittsburgh sample were reported to be taking medications at the time of scanning, but otherwise psychostimulants were withheld at least 24 hours prior to scanning. Subjects with ADHD are also known to have higher rates of head motion during MRI scanning and this has been proposed as a genuine trait, and perhaps genetic, difference (Couvy-Duchesne et al., 2016). Group differences in DVARS, the frame-to-frame measure of head motion, were significant at *p* < 0.05, with ADHD subjects having more head motion. Overall, our desire was to fashion a subject sample resembling clinical populations; therefore, we erred on the side of retaining differences in motion, handedness, comorbidities, and medication usage, and modeling these variables statistically. However, since the attribution of head motion differences to a trait difference in ADHD is not a settled question, we also created a subsample of 444 subjects (222 with ADHD and 222 with TD) that were further matched for head motion, quantified as DVARS score. The subjects that were omitted from the original sample to create this motion-matched subsample are detailed in Supporting Information Table S4 (de Lacy & Calhoun, 2019) Using a two-sample *t* test, we verified there was no significant difference in DVARS between subjects with ADHD and TD in the motion-matched subsample (*p* = 0.44). All methods detailed below were applied similarly to each subject sample, with any exceptions noted.

### Group Spatial Independent Component Analysis

After processing the fMRI data, we performed ICA using the Group ICA of fMRI Toolbox (GIFT) developed in our group and widely used in ICA of fMRI (Calhoun & Adali, 2012; Calhoun, Adali, Pearlson, & Pekar, 2001), using an established pipeline (Allen et al., 2011). Resting-state scans were first prewhitened and despiked (using 3dDespike) followed by a subject-specific data reduction principal component analysis retaining 63 principle components with the objective of stabilizing back reconstruction and retaining maximum variance at the individual level (Erhardt et al., 2011). A relatively high-order 60-component group ICA was then performed using the Infomax algorithm with best run selection from 10 randomly initialized analyses (Himberg, Hyvarinen, & Esposito, 2004; Li, Adali, & Calhoun, 2007). Aggregate spatial maps were estimated as the centrotypes of component clusters to reduce sensitivity to initial algorithm parameters. Single-subject images were concatenated in time to perform the single-group ICA estimation and subject-specific spatial maps estimated using back reconstruction (Erhardt et al., 2011) with the group information–guided ICA (GIG-ICA) algorithm (Du et al., 2016), an approach which we have shown captures individual subject variability well (Allen, Erhardt, Wei, Eichele, & Calhoun, 2012). GIG-ICA estimates single-subject images and time courses from the single (all subjects) group ICA estimation, thereby allowing individual variation in spatial maps. The resulting independent components were scaled by converting each subject component image and timecourse to z-scores.

### Sorting Components from the Spatial ICA

As is standard in ICA analysis, components were sorted using a semimanual process into two groups: (1) gray matter networks and (2) artifactual noise components. All gray matter networks were included in the study, and all noise components such as the effects of blood pulsations and motion were discarded. Sorting was performed using a combination of expert visual inspection and quantitative metrics. For each of the 60 components we computed the spectral metrics of (1) fractional amplitude of low-frequency fluctuations (fALFF) and (2) dynamic range (Allen et al., 2011). fALFF is the ratio of the integral of spectral power below 0.10 Hz to the integral of power between 0.15 and 0.25 Hz. Dynamic range is the difference between the peak power and minimum power at frequencies to the right of the peak. Generally, components representing brain networks have higher values in these spectral metrics, whereas noise components (such as signals accruing from cerebrospinal fluid, vascular pulsations, white matter, or head motion) have lower values, although there are currently no absolute cutoff points for inclusion or exclusion. Components were visually inspected by both authors to determine their correspondence with gray matter. Components with poor overlap with cerebral gray matter or low spectral metrics were discarded. The remaining 44 components represented the intrinsic networks (INs) used in this study, and the coordinates in Montreal Neurologic space associated with their top three peak intensities may be inspected in Supporting Information Table S5 (de Lacy & Calhoun, 2019). Discarded artifactual components may be inspected in Supporting Information Table S6 (de Lacy & Calhoun, 2019).

### Construction of Intrinsic Network Spatial Maps

**Figure 5.**
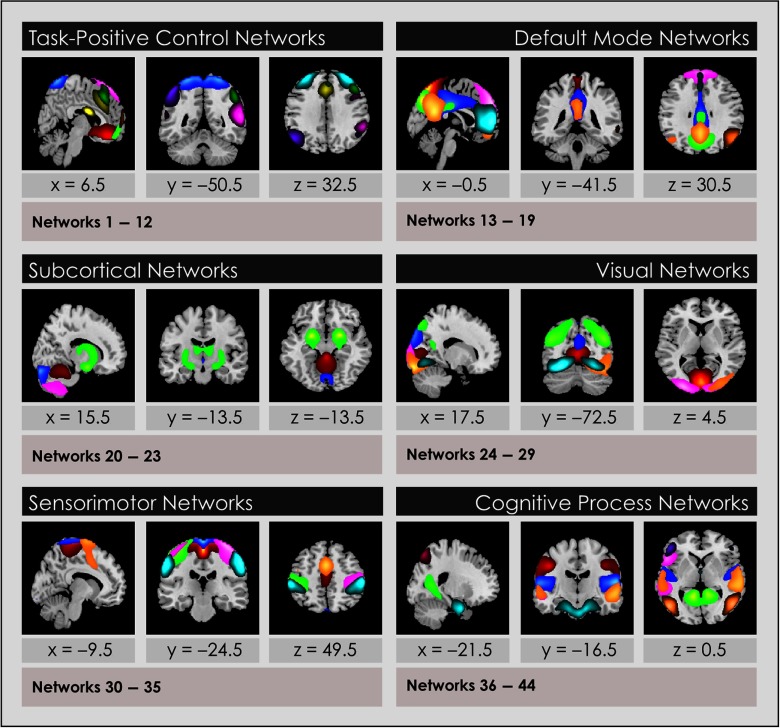
Intrinsic networks grouped by associated neurocognitive function. Forty-four functional networks were identified and analyzed from whole-brain resting-state functional MRI data in 504 age- and gender-matched subjects 7–17 years old: 252 typically developing youth and 252 with ADHD. Networks are shown grouped into domains. Top constituent anatomic regions for each network and an attribution of its function may be inspected in Supporting Information Table S1 (de Lacy & Calhoun, 2019).

We constructed network spatial maps by selecting voxels that represented the strongest and most consistent coactivations for each of the 44 gray matter INs included after the sorting process by performing a voxelwise one-sample *t* test on the individual subject time courses and thresholding individual voxels at (mean + 4 *SD*), again following an established pipeline (Allen et al., 2011) using GIFT. Thus, these spatial maps represent the brain regions most associated with each component’s time course, instantiated in thresholded brain maps. This procedure enabled us to construct a group spatial map for each of the networks assembled from the relevant individual subject time courses. These spatial maps were used to attribute the neurocognitive labels for each IN, and served as the inputs for the sFNC analysis to construct the sFNC matrices (Figure 5).

### Functional Intrinsic Network Attribution and Grouping

The primary neurocognitive function of each IN spatial map was attributed by visual inspection and quantitative comparisons by using three methods. First, we determined the coordinates in Montreal Neurologic Space (MNI) associated with peak intensities for each of the 44 INs. The top three coordinates were compared with the literature. We found multiple literature-based confirmatory sources that gave specific Talairach or MNI coordinates and associated these with network labels for all networks in the task-positive network group, the DMN, and primary sensorimotor and visual networks (Dosenbach et al., 2007; Dosenbach et al., 2006; Fox et al., 2005; Laird et al., 2011; Seeley et al., 2007; Smith et al., 2009; Spreng, Sepulcre, Turner, Stevens, & Schacter, 2013; Vernet, Quentin, Chanes, Mitsumasu, & Valero-Cabre, 2014), but not for INs in the subcortical or cognitive processing groups. Second, the neurocognitive function of the top five spatial locations in each IN were examined using the Brodmann Interactive Atlas (http://www.fmriconsulting.com/brodmann/Interact.html). Third, network correlations with reverse inference maps of regional activations associated with specific neurocognitive functions were inspected in Neurosynth (Yarkoni, Poldrack, Nichols, Van Essen, & Wager, 2011).

### Static Functional Network Connectivity Analysis

We performed a multivariate analysis of covariance (MANCOVA) using the MANCOVAN toolbox in GIFT to compare the effects of age with other possible predictors of variance in the same set of network maps for (1) all 504 subjects, (2) the 252 TD subjects, and (3) the 252 subjects with ADHD using an established method (Allen et al., 2011). To optimize for the large dimensions of the data, but enable statistical testing at each voxel, predictors were submitted to the MANCOVA with an *F* test at each iteration to produce a final reduced model for each outcome measure and network before univariate testing of significant predictors was performed on the original model. Nuisance regressors composed of individual sites, DVARS measure, and the six realignment parameters and their six first derivatives were regressed from the analysis by using the general linear model, prior to computing Pearson correlations between IN spatial maps. Each site was modeled as an individual regressor. We used age, gender, FSIQ-level, scan site, and DVARS measure as predictors for all three analyses. For the first analysis of the combined group of 504 subjects, group or diagnosis of ADHD was also added as a predictor. Although we did remove the effects of DVARS measure and site prior to computing FNC matrices, we retained these as predictors in the MANCOVA to test for any residual effects of motion or site on results obtained in the statistical testing. For example, we tested for group × DVARs interactions. Significant effects were computed for both positively correlated voxels in each network and for voxels with anticorrelated time courses. We corrected for FDR at α = 0.01 for the 504-subject model and α = 0.05 for the smaller models of 252 subjects, given their reduced power.

To assess for possible effects of handedness, presence of comorbid diagnosis, and medication usage, we constructed an additional multivariate analysis in the original ADHD group of 252 subjects, where sensitivity to these effects would be larger than if diluted by the addition of TD controls. We used age, gender, FSIQ-level, scan site, DVARS, handedness, presence of comorbid diagnosis, and medication usage as predictors and otherwise duplicated our multivariate methodology. Comprehensive meta-analysis of fMRI studies suggests that ADHD-related dysfunction is present regardless of comorbid psychiatric conditions or history of stimulant treatment (Cortese et al., 2012).

### Computation of Brain States in Native State Space

To identify dynamic brain states, we adopted the framework (Allen et al., 2014; Sakoglu et al., 2010) of deriving a small number of stable FNC states from fMRI time courses by applying a clustering algorithm to a succession of FNC windows. First, subject time courses were detrended and despiked to remove outliers by using 3dDespike in the AFNI software, and filtered using a fifth-order Butterworth low-pass filter with a high-frequency cutoff of 0.15 Hz. After regression of six head motion parameters and their first temporal derivatives from the time courses, windowed covariance matrices were assembled using a sliding window approach instantiated in the temporal dFNC toolbox in GIFT, where a tapered rectangular window was created by convolving a rectangle (width = 25 TRs) with a Gaussian and slid in steps of 1 TR. Windowed FNC covariance matrices (wFNC) were estimated using a graphical LASSO method (Friedman, Hastie, & Tibshirani, 2008) as detailed in (Allen et al., 2014). The window size was selected based on previous studies demonstrating window sizes in the range of 40–60 s, which produce reasonable and robust results (Allen et al., 2014; Damaraju et al., 2014; de Lacy et al., 2017). Since this is multisite data, the TR varies among the four sites with a range of 1.5 to 2.5 s, and our algorithm incorporated this. Successive wFNC were then concatenated to form an array representing a state transition vector, or how the FNC state changed as a function of time for each subject. Subsequently, the *k*-means algorithm with city distance function was applied to this state transition vector to derive stable dynamic states, initializing the clustering of data from all subjects with cluster centroids that were first clustered using a subset of windows with local maxima in FC variance as in Allen et al. (2014) followed by clustering of the entire set of windows after initialization with the previous cluster solution. The subsampling procedure was performed to improve performance and reduce computational demand; previous results show that the solution improves results (Allen et al., 2014). We computed the number of clusters in the data by using the Elbow criterion and the Bayes information criterion (Supporting Information Figure S1, de Lacy & Calhoun, 2019). The former produced a cluster number of four, and the latter of six. We presented the average solution of five clusters, but also reproduced the analyses using the same methods for four- and six-cluster solutions and report all results throughout. In addition, we applied a threshold concept requiring that a given FNC covariance matrix be present in a minimum number of 10 windows for each subject included. Finally, we also performed a sensitivity analysis on the window size for the global 44-network dynamic models, analyzing each of the 4-, 5-, and 6-state solutions with window sizes of 20TR and 30TR. Generally, our framework fixes the number of clusters for all subjects since we believe allowing cluster number to vary among subjects could introduce model bias, since mathematically the results of computations in secondary metrics could be influenced by differences in cluster number. For example, it may be appreciated that comparing the fraction of total scan time two subjects spent in a state might differ for spurious reasons if one subject was moving among five clusters (states), and the other among four. Instead, our approach allows for potential differences in “dynamic biology” by permitting subjects to not enter (be present in) any given cluster. We computed the number of subjects from each group (TD vs. ADHD) in each state for every permutation of the analysis. Although these were in some cases different, this difference did not reach statistical significance tested at α < 0.05, corrected for FDR. In the motion-matched sample, we performed similar procedures for a five-cluster analysis with window size of 25TR, representing the primary analytic parameters presented for the original sample.

### Measures of Dynamism in Native State Space

We computed four measures of functional dynamism in the native state space, that is, on the stable brain states computed with *k* means. These included three measures of fluidity: the number of times each subject moved between these states during their individual time courses, the average time (in windows) they spent in each of the states once entering that state (dwell time), and the fraction of their total time course spent in each state. We also computed FNC between pairwise INs within each state.

### Computation of Prototype Connectivity Patterns in Meta-State Space

To create prototype connectivity patterns (CPs) for use in higher dimensional measures of dynamism, we followed a similar method but in this case preferred the use of the tICA algorithm (Miller et al., 2016). After creating wFNC series as detailed above, the tICA algorithm was applied to the individual arrays of FNC covariance matrices by using the city method and the algorithm iterated a maximum of 200 times before convergence. We chose the tICA algorithm since its decomposition produces CPs whose weights in the wFNC are maximally temporally mutually independent. However, we have previously demonstrated that results using our dynamic measures (below) are stable if other clustering measures such as *k* means, spatial ICA, and principal component analysis are used (Miller et al., 2016). CPs formed the analytic substrate for the remainder of the study.

### High-Dimension Measures of Dynamism in Meta-State Space

We computed four measures of dynamism in the higher dimensional meta-state space by using the same procedure as detailed by Miller and colleagues (Miller, Yaesoubi, & Calhoun, 2014; Miller et al., 2016). Here, the time-varying, additive contributions made by CPs to each observed wFNC over the subject time courses are discretized. A five-dimensional weight vector is obtained representing the contribution of each CP to each wFNC matrix by regressing the FNC estimate onto the tICA cluster centroid. Real-valued weights accruing from this computation are then replaced by a value in ± (1, 2, 3, 4) according to the signed quartile into which each weight falls. The resulting discretized vectors are termed meta-states. Four measures of dynamism were computed for these meta-states. Discretization of the CP contributions to wFNC constructs a state space comprised of 8^5^ = 32,768 possible meta-states (and similarly, 8^4^ and 8^6^ possible states in the sensitivity analysis), which subjects may occupy over time. Two metrics describe the fluidity with which subjects traverse the meta-state space: the number of distinct meta-states passed through by each individual and the number of times each subject switches between meta-states. The remaining two metrics describe the high-dimension dynamic range achieved by subjects: the maximal L^1^ span achieved between occupied meta-states, and the total distance “traveled” by an individual through the state space (sum of all L^1^ distances).

### Local and Global Measures of Dynamism

In this work, we introduce the concept of local versus global analysis of brain dynamism as observed in fMRI. Here, we applied the concepts of dFNC and meta-state analysis not only to the global set of INs but also to subsets of INs grouped by neurocognitive attribution. The formation of a high-order connectivity model with 44 networks is particularly tractable for this approach since it allows the examination of dynamism among subnetworks of the same system. In this procedure, the above pipeline was repeated in the same fashion for (1) the overall group of 44 networks, (2) within each of the six groups of INs when grouped by their dominant neurocognitive attribution (e.g., among 7 subnetworks of the default mode system), and (3) between the default mode system and each of the other functional system groups (e.g., among the default mode subnetworks and the sensorimotor networks).

### Dynamic Statistical Analysis

Group differences in each measure of dynamic connectivity between TD subjects and subjects with ADHD were calculated using two-sample *t* tests. These were computed at a significance level of α < 0.05, corrected to control FDR where multiple comparisons obtained. We performed this analysis using the general linear model to disambiguate the effects of maturation (age) versus group effect of ADHD > TD. Variance associated with site, DVARS measure, IQ-level, and gender was removed by regression using the general linear model from the correlation values prior to performing clustering. The analysis was then repeated, removing variance associated with site, DVARS measure, IQ-level, gender, and age using the general linear model.

## ACKNOWLEDGMENTS

The authors would like to thank B. Ernesto Johnson for his assistance with figure preparation.

## AUTHOR CONTRIBUTIONS

Nina de Lacy: Conceptualization; Formal analysis; Investigation; Writing – original draft. Vince Calhoun: Supervision; Writing – review & editing.

## FUNDING INFORMATION

Nina de Lacy, National Center for Advancing Translational Sciences (http://dx.doi.org/10.13039/100006108), Award ID: KL2TR000421 and KL2TR002317. Vince Calhoun, National Institutes of Health (http://dx.doi.org/10.13039/100000002), Award ID: 2R01EB005846. Vince Calhoun, National Institutes of Health (http://dx.doi.org/10.13039/100000002), Award ID: P20GM103472. Vince Calhoun, National Institutes of Health (http://dx.doi.org/10.13039/100000002), Award ID: R01EB020407. Vince Calhoun, National Science Foundation (http://dx.doi.org/10.13039/100000001), Award ID: 153906.

## TECHNICAL TERMS

Intrinsic connectivity: Refers to functional connectivity as measured during task-free states such as the wakeful resting state. It is inferred that this represents the spontaneous, or intrinsic, activity of the brain.
Segregation: Refers to brain regions or networks that exhibit anti-correlated functional connectivity.
L1 span: Refers to the maximal L1 distance achieved between occupied meta-states.
L1 distance: Refers to the L1 norm or Minkowski distance which is a metric that computes the distance between two points as the sum of the absolute differences of their Cartesian coordinates.
Functional connectivity: Refers to the strength of connections between brain regions or networks that share neurocognitive, or functional, properties. More specifically, temporal correlation is assessed using statistical methods. Regions or networks may exhibit positively correlated or negatively correlated (anti-correlated) relationships.
Dynamic connectivity: Refers to the phenomenon of functional connectivity changing over time. Analysis of this time-varying connectivity is commonly performed using functional MRI, but the phenomenon has been observed using other experimental modalities.
Scrubbing: Is a method used in functional MRI to address unwanted ’noise’ accruing from head otion during the acquisition of sequential MRI volumes. Individual volumes where large head motion is present are removed from the signal.
