# Supplementary material for: Dynamic connectivity and the effects of maturation in youth with attention deficit hyperactivity disorder
Source: Netw Neurosci. 2018 Dec 1;3(1):195–216. doi: 10.1162/netn_a_00063 (PMC6372020; doi:10.1162/netn_a_00063)
Supplement: Supplementary file 1 [file netn-03-195-s001.pdf]

Supplementary Table 1

de Lacy, N., & Calhoun, V. D. (2019). Dynamic connectivity and the effects of maturation in youth with attention deficit hyperactivity disorder. *Network Neuroscience*. *3*(1), 195–216. https://doi.org/10.1162/netn_a_00063

	Native Space			Meta-State Space			
**IN Group**	**Dwell Time**	**Fraction of Time**	**State-switching**	**Number of states**	**State-switching**	**L1 Span**	**Total L1 Distance**
**Global**	0.210-0.354	0.870	0.230	0.030	0.060	0.020	0.093
**Task-positive Control**	0.060-0.765	0.353-0.852	0.229	0.063	0.054	0.110	0.059
**DMN**	0.007-0.961	0.132-0.957	0.297	0.417	0.354	0.306	0.322
**Subcortical**	0.276-0.838	0.787	0.946	0.307	0.256	0.835	0.429
**Visual**	0.219-0.985	0.652-0.890	0.708	0.703	0.718	0.233	0.530
**Sensorimotor**	0.062-0.875	0.738	0.136	0.629	0.874	0.446	0.155
**Cognitive Processing**	0.343-0.921	0.779	0.674	0.897	0.849	0.866	0.235

Table 1: Global and local intra-system dynamic fluidity and range measures in subjects with ADHD and TD

Statistical significance level (p-value or a-value) is displayed for differences in ADHD>TD for each measure of fluid dynamism in the 5-cluster analyses for global and within-system, network groupings. These values represent results obtained in the subsample of 444 youth, matched for motion (DVARS), after variance attributable to age was removed using the general linear model. Significant ($p<0.05$ or $a<0.05$, corrected for FDR) differences are highlighted in blue (ADHD<TD) and red (ADHD>TD). Dwell time and fraction of time p-values are shown in a range where applicable, indicating the lowest and highest p-values in all 5 states. The DMN was the only system in which dwell time was significant, in only one state represented by the specific lower bound p-value shown.

Supplementary Table 2

	Native Space			Meta-State Space			
IN Groups	Dwell Time	Fraction of Time	State transitions	Number of states	State-switching	L1 Span	Total L1 Distance
**DMN x Task-positive control**	0.287-0.924	0.873	0.119	0.040	0.036	0.128	0.229
**DMN x Sensorimotor**	0.143-0.813	0.644	0.786	0.300	0.400	0.931	0.462
**DMN x Visual**	0.283-0.969	0.883	0.986	0.534	0.554	0.963	0.373
**DMN x Subcortical**	0.097-0.788	0.734-0.963	0.034	0.627	0.428	0.165	0.807
**DMN x Cognitive Processing**	0.354-0.874	0.587	0.495	0.939	0.954	0.645	0.604

Table 2: Local DMN inter-system dynamic fluidity and range measures in subjects with ADHD and TD

Statistical significance level (p-value or a-value) is displayed for differences in ADHD>TD for each measure of fluid dynamism in the 5-cluster analyses for cross-system grouping between the DMN group and every other network group. All values represent 2-sample t-tests performed at $p<0.05$ or $a<0.05$, corrected for FDR. These values represent results obtained in the subsample of 444 youth, matched for motion (DVARS), after variance attributable to age was removed using the general linear model. Significant ($p<0.05$ or $a<0.05$, corrected for FDR) differences are highlighted in red (ADHD>TD). Dwell time and fraction of time values are shown in a range where applicable, indicating the lowest and highest bounds for each of the 5 states.

Supplementary Table 3

The following is a list of subjects included in the study, with their ID codes as given in the ADHD-200 repository.

Typically-developing:

10006	16008	25013	1842819	2558999	3385520	5600820
10009	16009	1000804	1849382	2559537	3434578	5669389
10010	16010	1018959	1854691	2572285	3473830	6187322
10021	16012	1043241	1854959	2618929	3494778	6346605
10023	16021	1050345	1875084	2640795	3554582	6477085
10024	16026	1050975	1875711	2641332	3562883	6550938
10036	16027	1056121	1879542	2659769	3593327	7011503
10039	16028	1093743	1884448	2703336	3605062	7093319
10043	16029	1117299	1916266	2714224	3610134	7129258
10045	16030	1127915	1934623	2730704	3624598	7135128
10046	16031	1177160	1985430	2735617	3655623	7407032
10052	16033	1245758	1995121	2822304	3662296	7994085
10054	16035	1253411	2018106	2833684	3672854	8083695
10057	16036	1258069	2033178	2872641	3699991	8191384
10058	16037	1266183	2051479	2903997	3707771	8263351
10059	16038	1302449	2101067	2917777	3732101	8692452
10063	16039	1320247	2106109	2930625	3809753	8834383
10068	16044	1359325	2107404	2991307	3813783	9221927
10069	16050	1404738	2136051	3004580	3845761	9578631
10070	16052	1408093	2140063	3086074	3884955	9578663
10076	16055	1411536	2208591	3103809	3889095	9640133
10079	16060	1419103	2249443	3107623	3902469	9750701
10082	16061	1435954	2266806	3157406	3912996	9783279
10088	16063	1494102	2268253	3163200	3930512	9887336
10092	16064	1517058	2275786	3212536	3972956	9922944
10099	20014	1562298	2310449	3224401	3993793	
10100	20015	1567356	2377207	3233028	4048810	
10102	20021	1581470	2411995	3239413	4053836	
10110	21005	1638334	2443191	3243657	4079254	
10112	21007	1662160	2488729	3248920	4084645	
10114	21016	1686092	2493190	3262042	4125514	
10117	21019	1686265	2498847	3269608	4136226	
10122	21029	1689948	2511886	3277313	4154182	
10123	21034	1692275	2528407	3306863	4164316	
10125	21038	1700637	2535087	3308331	4265987	
10128	25000	1737393	2538839	3320367	4383707	
16004	25003	1779922	2554127	3348989	4921428	
16006	25009	1794770		3349423	5575344	

Subjects with ADHD:

10001	10096	25008	2026113	2984158	3983607
10002	10101	25012	2030383	2996531	4006710
10007	10104	25014	2031422	3119327	4028266
10011	10106	1019436	2054438	3124419	4053388
10012	10107	1023964	2081148	3154996	4055710
10013	10108	1038415	2107638	3160561	4060823
10017	10109	1057962	2141250	3169448	4073815
10019	10115	1094669	2174595	3170319	4075719
10020	10116	1099481	2196753	3174224	4091983
10022	10118	1133221	2207418	3194757	4095229
10026	10126	1159908	2228148	3205761	4095748
10028	10129	1186237	2230510	3235580	4116166
10029	20008	1187766	2260910	3291029	4154672
10030	20010	1201251	2276801	3313497	4187857
10032	21002	1208795	2297413	3349205	4221029
10033	21003	1240299	2306976	3378296	4241194
10035	21006	1282248	2380326	3390312	4275075
10037	21008	1283494	2380967	3407871	4334113
10040	21009	1341865	2497695	3433846	4362730
10041	21010	1356553	2505328	3441455	5150328
10042	21013	1399863	2524687	3446674	5164727
10047	21014	1471736	2529026	3457975	5193577
10048	21015	1497055	2570769	3504058	5971050
10050	21017	1511464	2599965	3519022	5993008
10051	21021	1517240	2601519	3520880	6383713
10056	21022	1561488	2628237	3542588	6453038
10060	21023	1623716	2682736	3559087	6500128
10061	21025	1628610	2697768	3561920	7253183
10062	21026	1643780	2737106	3601861	7390867
10064	21027	1740607	2741068	3653737	7591533
10071	21030	1771270	2773205	3672300	7689953
10073	21031	1780174	2780647	3679455	8009688
10074	21032	1784368	2821683	3691107	8278680
10075	21033	1791543	2854839	3712305	8337695
10078	21035	1809715	2884672	3767334	8463326
10081	21036	1843546	2897046	3803759	8697774
10085	21040	1883688	2907951	3827352	8915162
10086	21042	1918630	2910270	3834703	9002207
10087	21043	1947991	2919220	3856956	9190596
10090	21044	1996183	2940712	3870624	9210521
10091	21046	2014113	2950672	3910672	9326955
10095	25002	2024999	2950754	3976121	9907452

Supplementary Table 4

The following is a list of subjects that were omitted from the original sample of 504 youth, to create a sub-sample of 444 subjects that were additionally matched for motion. ID codes are listed as given in the ADHD-200 repository. There was no significant difference in motion, as calibrated by DVARS score, between the two groups (p=0.44).

Typically-Developing	ADHD
20021	10012
25009	10013
1050345	10028
1517058	10056
1686092	10061
1854691	10062
1879542	10074
1985430	10085
2033178	10096
2101067	10106
2268253	20008
2488729	20010
3224401	1019436
3233028	1099481
3277313	1283494
3306863	1471736
3308331	1628610
3605062	1780174
3624598	1809715
3655623	2014113
3732101	2081148
4048810	2174595
4136226	2682736
4383707	3170319
5575344	3390312
5600820	4362730
7135128	6453038
7407032	8009688
7994085	8337695
9783279	8915162

Supplementary Table 5

44 individual brain networks identified from group ICA. Co-ordinates in Montreal Neurologic Space are displayed for the top 3 maxima for each network. For each co-ordinate set, peak intensities for the left brain are shown first, followed by right brain co-ordinates. The attribution of each network by domain and for each specific network are also listed.

Domain	Network Attribution	Number	Top 3 Positively-Correlated Intensities
Task-Positive	Ventral Attention: ventral frontal	1	(-3, 38, -10)/(3, 38, -10)<br>(-6, 41, -10)/(3, 35, -13)<br>(-9, 23, -13)/(3, 23, -13)
Task-Positive	Dorsal Attention	2	(-12, -58, 65)/(9, -58, 65)<br>(-18, -55, 65)/(15, -61, 62)<br>(-9, -58, 62)/(9, -61, 62)
Task-Positive	Orbitofrontal	3	(-42, 47, -13)/(45, 50, -13)<br>(-48, 29, -13)/(48, 35, -13)<br>(-33, 53, -16)/(21, 56, -13)
Task-Positive	Dorsomedial Prefrontal	4	(-12, 47, 53)/(36, 23, 56)<br>(-27, 23, 62)/(42, 26, 53)<br>(-3, 50, 47)/(0, 0, 0)
Task-Positive	Cingulo-opercular: insula	5	(-39, 11, -1)/(42, 11, -4)<br>(-39, 17, -4)/(42, 17, -4)<br>(-45, 2, -4)/(48, 8, -7)
Task-Positive	Dorsolateral prefrontal	6	(-30, 50, 23)/(30, 47, 23)<br>(-30, 53, 20)/(30, 47, 29)<br>(-3, 29, 32)/(3, 29, 32)
Task-Positive	Frontopolar/dorsolateral prefrontal	7	(-30, 56, 11)/(33, 62, 5)<br>(-27, 56, 8)/(30, 65, 8)<br>(-42, 50, 5)/(42, 59, 5)
Task-Positive	Temporo-limbic	8	(-42, 14, -19)/(45, 14, -16)<br>(-42, 17, -16)/(39, 17, -16)<br>(-18, -1, -22)/(18, -1, -22)
Task-Positive	Left fronto-parietal control	9	(-45, -67, 38)/(51, -55, 41)<br>(-48, -61, 35)/(51, -61, 35)<br>(-51, -61, 32)/(51, -52, 38)
Task-Positive	Cingulo-opercular: anterior cingulate	10	(-36, 20, -4)/(36, 20, -7)<br>(-3, 23, 38)/(3, 26, 35)<br>(-3, 35, 29)/(6, 32, 29)
Task-Positive	Ventral attention: temporoparietal junction	11	(-60, -52, 14)/(54, -46, 17)<br>(-60, -43, 26)/(54, -46, 23)<br>(0, 0, 0)/(57, -49, 23)
Task-Positive	Right fronto-parietal control	12	(0, 0, 0)/(30, 53, 2)<br>(0, 0, 0)/(27, 56, 2)<br>(0, 0, 0)/(39, 53, 5)
Default Mode	Posterior: precuneus	13	(-3, -64, 50)/(3, -67, 59)<br>(-9, -67, 56)/(9, -67, 59)<br>(0, -46, 53)/(3, -46, 50)
Default Mode	Posterior: posterior cingulate	14	(-3, -31, 35)/(3, -40, 26)<br>(-3, -43, 23)/(3, -43, 23)<br>(0, -28, 47)/(3, -25, 47)
Default Mode	Posterior: precuneus	15	(-6, -73, 35)/(6, -73, 38)<br>(-6, -70, 32)/(12, -70, 32)<br>(-3, -25, 29)/(3, -64, 26)
Default Mode	Anterior: medial prefrontal	16	(-3, 56, 29)/(6, 56, 29)<br>(-3, 50, 26)/(3, 50, 26)<br>(-6, 44, 17)/(3, 47, 11)
Default Mode	Posterior: posterior cingulate/precuneus	17	(-3, -61, 26)/(6, -52, 29)<br>(-3, -55, 32)/(3, -55, 32)<br>(-6, -61, 23)/(6, -52, 23)

Default Mode	Anterior: medial prefrontal/anterior cingulate	18	(-3, 50, 2)/(3, 50, 2)<br>(-3, 53, 5)/(3, 53, 5)<br>(-6, 59, 2)/(6, 59, 2)
Default Mode	Posterior: posterior cingulate	19	(0, 0, 0)/(48, -67, 35)<br>(-42, -70, 38)/(51, -67, 38)<br>(0, 0, 0)/(51, -58, 32)
Subcortical	Cerebellum	20	(-6, -46, -19)/(9, -46, -19)<br>(-9, -49, -19)/(12, -46, -22)<br>(-6, -58, -19)/(12, -55, -19)
Subcortical	Cortico-cerebellar	21	(-15, -85, -31)/(36, -76, -31)<br>(-27, -82, -37)/(39, -76, -34)<br>(-24, -82, -34)/(36, -79, -34)
Subcortical	Basal Ganglia-Thalamic	22	(-18, 8, -1)/(21, 8, 2)<br>(-24, 2, -13)/(27, -4, -13)<br>(-18, 5, -13)/(21, 5, -13)
Subcortical	Striato-cerebellar	23	(-12, -61, -52)/(12, -64, -52)<br>(-15, -58, -52)/(12, -61, -49)<br>(0, 0, 0)/(15, -73, -43)
Visual	Visual	24	(-3, -85, 2)/(3, -85, 2)<br>(-3, -88, -1)/(3, -88, -1)<br>(-9, -70, 8)/(6, -70, 8)
Visual	Visual	25	(-6, -88, 26)/(3, -82, 29)<br>(0, -76, 26)/(3, -76, 23)<br>(-21, -94, 17)/(15, -94, 14)
Visual	Visual	26	(-36, -82, 26)/(39, -79, 26)<br>(-33, -79, 32)/(36, -76, 32)<br>(-30, -79, 35)/(33, -73, 35)
Visual	Visual	27	(-21, -100, 2)/(33, -94, -1)<br>(-18, -100, -1)/(27, -100, -4)<br>(-24, -97, -10)/(27, -100, -10)
Visual	Visual	28	(0, 0, 0)/(36, -82, -10)<br>(0, 0, 0)/(33, -82, -13)<br>(0, 0, 0)/(27, -85, -19)
Visual	Visual	29	(-24, -64, -10)/(27, -73, -13)<br>(-27, -61, -13)/(24, -70, -13)<br>(-27, -58, -10)/(24, -49, -13)
Sensorimotor	Sensorimotor: lower limb	30	(0, -22, 56)/(3, -19, 62)<br>(-21, -31, 68)/(21, -31, 68)<br>(-18, -34, 68)/(18, -28, 68)
Sensorimotor	Gesture: fusiform face area, motor cortex	31	(-3, -19, 71)/(3, -13, 71)<br>(-3, -43, 71)/(3, -37, 71)<br>(-3, -49, 68)/(6, -46, 71)
Sensorimotor	Left primary sensorimotor	32	(-45, -31, 53)/(45, -28, 53)<br>(-42, -34, 47)/(0, 0, 0)<br>(-33, -34, 62)/(0, 0, 0)
Sensorimotor	Right primary sensorimotor	33	(-45, -31, 56)/(51, -19, 50)<br>(-36, -31, 62)/(36, -31, 59)<br>(0, 0, 0)/(33, -31, 50)
Sensorimotor	Supplementary sensorimotor area	34	(0, 8, 50)/(3, 8, 47)<br>(0, 5, 53)/(3, 8, 53)<br>(-3, 11, 44)/(6, 8, 44)
Sensorimotor	Somatosensory-tactile	35	(-51, -31, 44)/(60, -19, 35)<br>(-60, -28, 35)/(60, -25, 35)<br>(-60, -22, 41)/(57, -19, 38)
Cognitive Processing	Primary speech	36	(-54, -10, 32)/(54, -7, 35)<br>(-60, -10, 17)/(60, -10, 17)<br>(-60, -13, 14)/(63, -10, 14)
Cognitive Processing	Auditory	37	(-45, -16, 8)/(48, -22, 14)<br>(-48, -16, 11)/(51, -16, 11)<br>(-48, -22, 11)/(48, -19, 11)

Cognitive Processing	Memory, perceptual: retrosplenial and parahippocampal	38	(-3, -67, 14)/(12, -55, 5)<br>(-6, -67, 17)/(9, -64, 17)<br>(-9, -61, 5)/(3, -73, 14)
Cognitive Processing	Language comprehension	39	(-45, 23, -7)/(0, 0, 0)<br>(-48, 17, -10)/(0, 0, 0)<br>(-51, 17, 8)/(0, 0, 0)
Cognitive Processing	Speech comprehension	40	(-57, -13, -10)/(54, -7, -13)<br>(-51, 5, -13)/(51, 11, -19)<br>(0, 0, 0)/(60, -7, -19)
Cognitive Processing	Episodic memory: hippocampal/temporo-limbic	41	(-21, -4, -25)/(24, -1, -25)<br>(-18, -4, -22)/(24, -4, -28)<br>(-24, -7, -25)/(30, -7, -25)
Cognitive Processing	Fusiform face area, bilateral	42	(-48, -67, 8)/(51, -64, -1)<br>(-48, -70, 5)/(51, -64, -7)<br>(-45, -67, -1)/(48, -67, -1)
Cognitive Processing	Spatial attention, calculation	43	(-24, -67, 47)/(30, -61, 50)<br>(-36, -55, 53)/(33, -61, 47)<br>(-24, -64, 50)/(27, -61, 53)
Cognitive Processing	Semantic processing, reading	44	(-48, 32, 17)/(54, 32, 23)<br>(-45, 32, 14)/(54, 29, 20)<br>(-42, 2, 38)/(0, 0, 0)

Supplementary Table 6

16 components were discarded after sorting of the 60 components from the group spatial ICA. Representative image slices are displayed for each of the discarded components along with possible functional attributions.

Representative Image Slice	#	Suggested attribution	Representative Image Slice	#	Suggested attribution
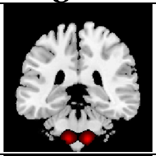	45	Brainstem pulsation	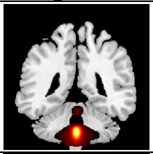	53	Vascular
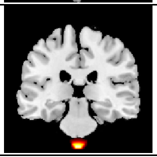	46	Brainstem pulsation	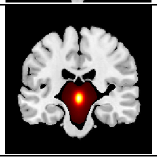	54	Ventricles
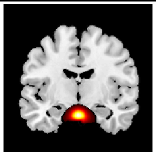	47	Vascular	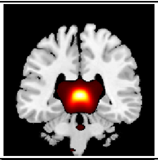	55	Ventricles
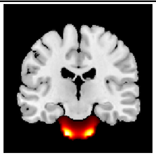	48	Brainstem pulsation	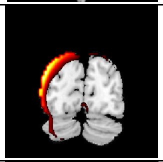	56	Motion
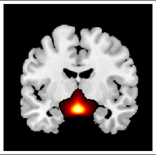	49	Vascular	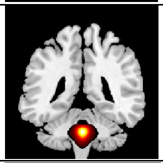	57	Vascular
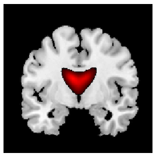	50	Ventricles	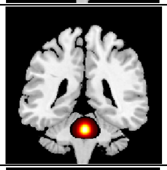	58	Vascular
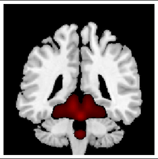	51	Ventricles	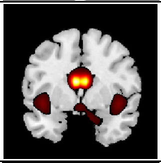	59	Ventricles/motion
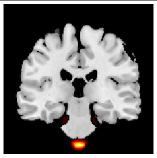	52	Brainstem pulsation	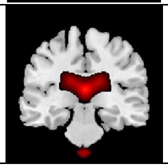	60	Ventricles/brainstem pulsation

Supplementary Figure 1

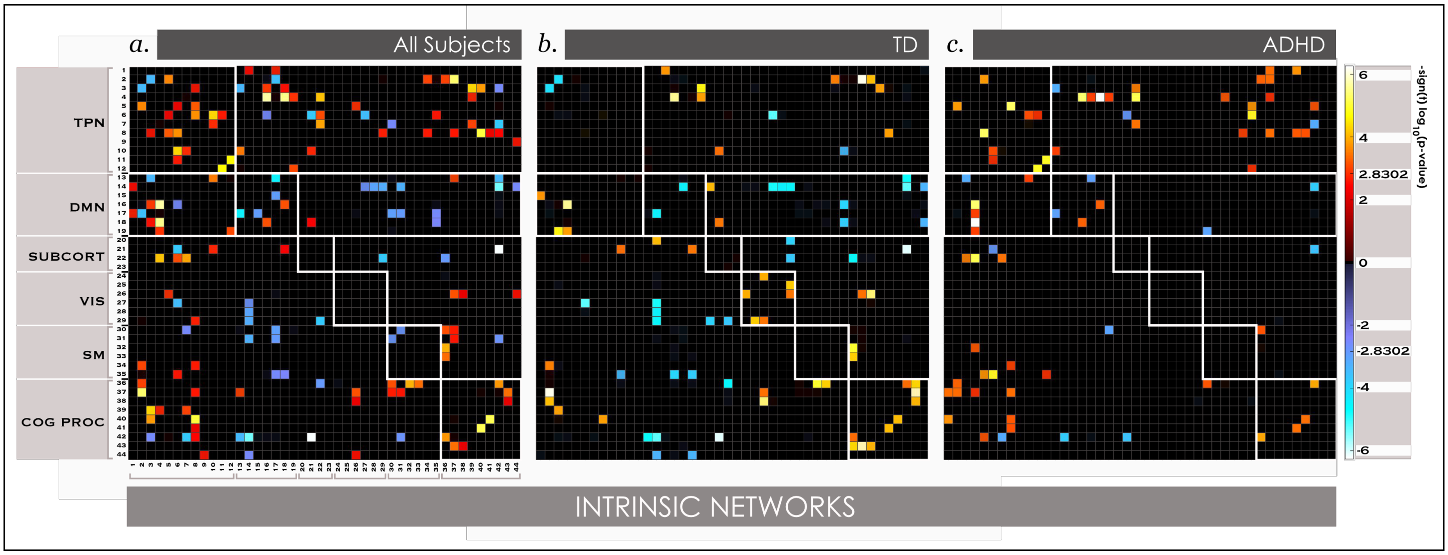

Age effects in static connectivity in 222 subjects with TD and 222 with ADHD ages 7-17 was determined by computing Pearson pairwise correlations averaged across fMRI timecourses among 44 INs obtained from ICA. These subjects were part of a subsample created that was matched for motion, as defined by DVARS. In **a**, significant (a<0.01corrected for FDR) age effects are displayed for all 444 subjects, where **b** and **c** show significant (a<0.05, corrected for FDR) age effects for TD and ADHD youth, respectively.

Supplementary Figure 2

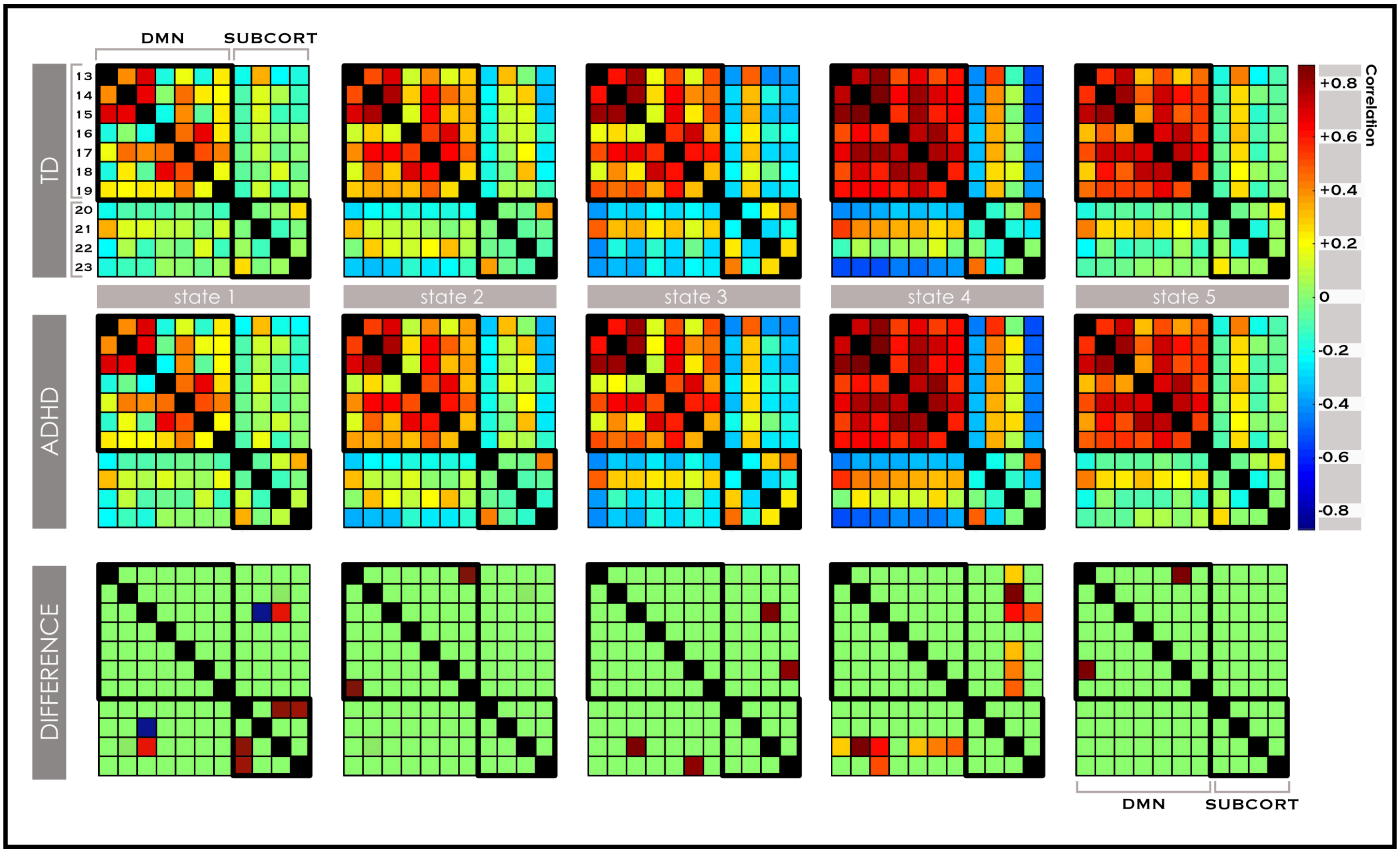

Dynamic functional connectivity across 5 brain states was estimated with k-means clustering of windowed FNC from the ICA timecourses in local intrinsic connectivity between DMN and subcortical networks for a subsample of 444 youth that were additionally matched for motion, defined by DVARS. Significant FNC differences between any combination of intrinsic network systems were examined with 2-sample t-tests at a significant level of a<0.05, corrected for false discovery rate.

Supplementary Figure 3

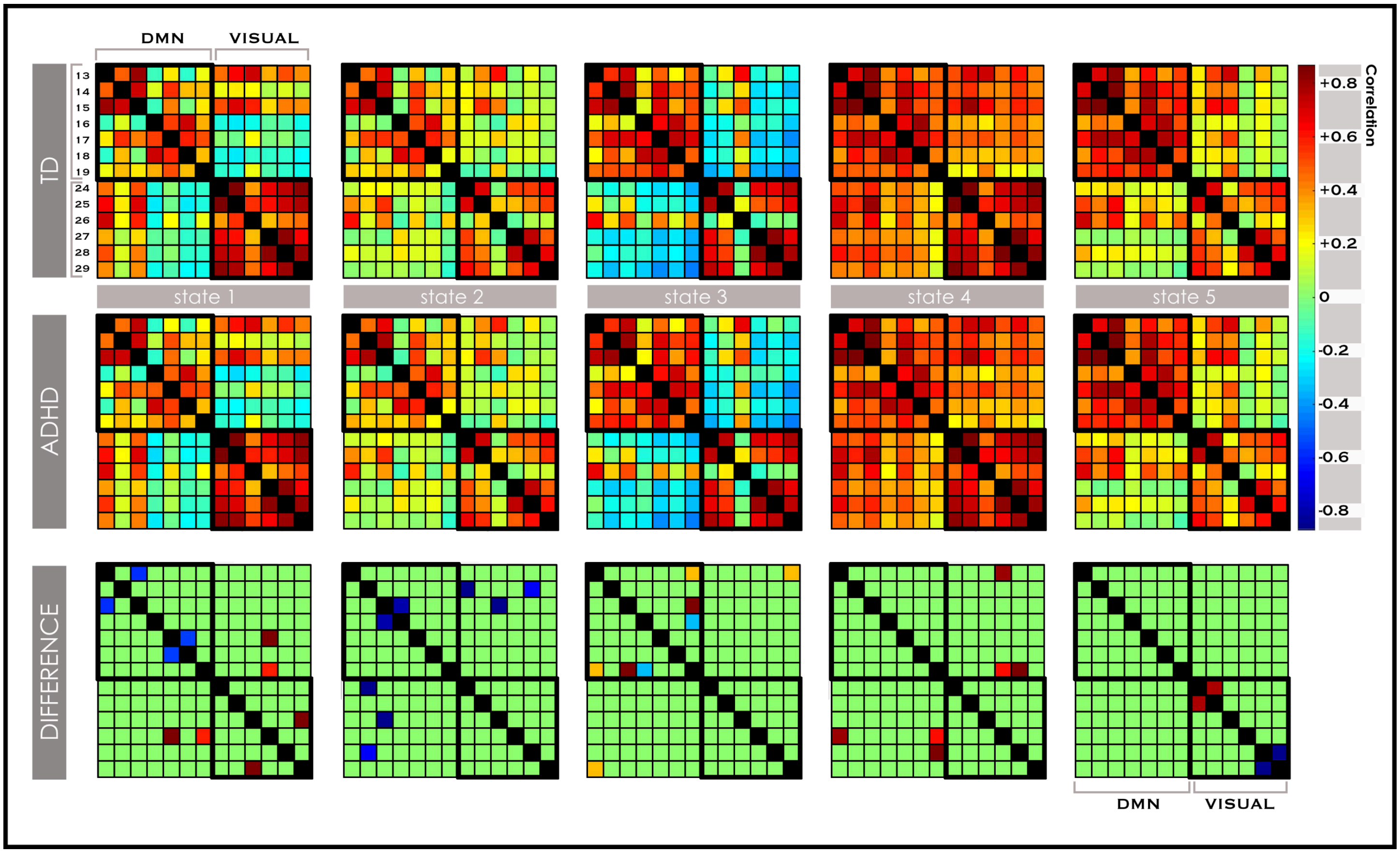

Dynamic functional connectivity across 5 brain states was estimated with k-means clustering of windowed FNC from the ICA timecourses in local intrinsic connectivity between DMN and visual networks for a subsample of 444 youth that were additionally matched for motion, defined by DVARS. Significant FNC differences between any combination of intrinsic network systems were examined with 2-sample t-tests at a significant level of α<0.05, corrected for false discovery rate.

Supplementary Figure 4

The number of clusters (states) in the dynamic connectivity analysis was determined using the Elbow Criterion and Bayes Information Criterion, log-likelihood method. Scree plots are displayed below for these computations on windowed FNC matrices including variance attributable to age for a) Elbow criterion and b) BIC, and after variance attributable to age was removed using the general linear model for c) Elbow criterion and d) BIC.

a)

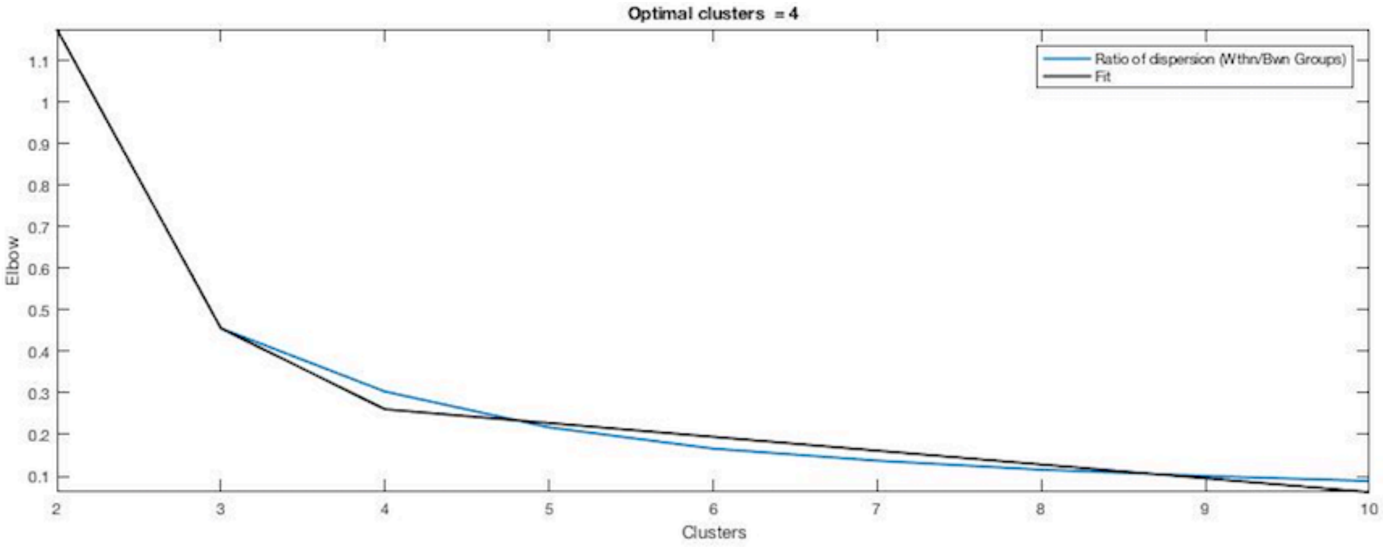

b)

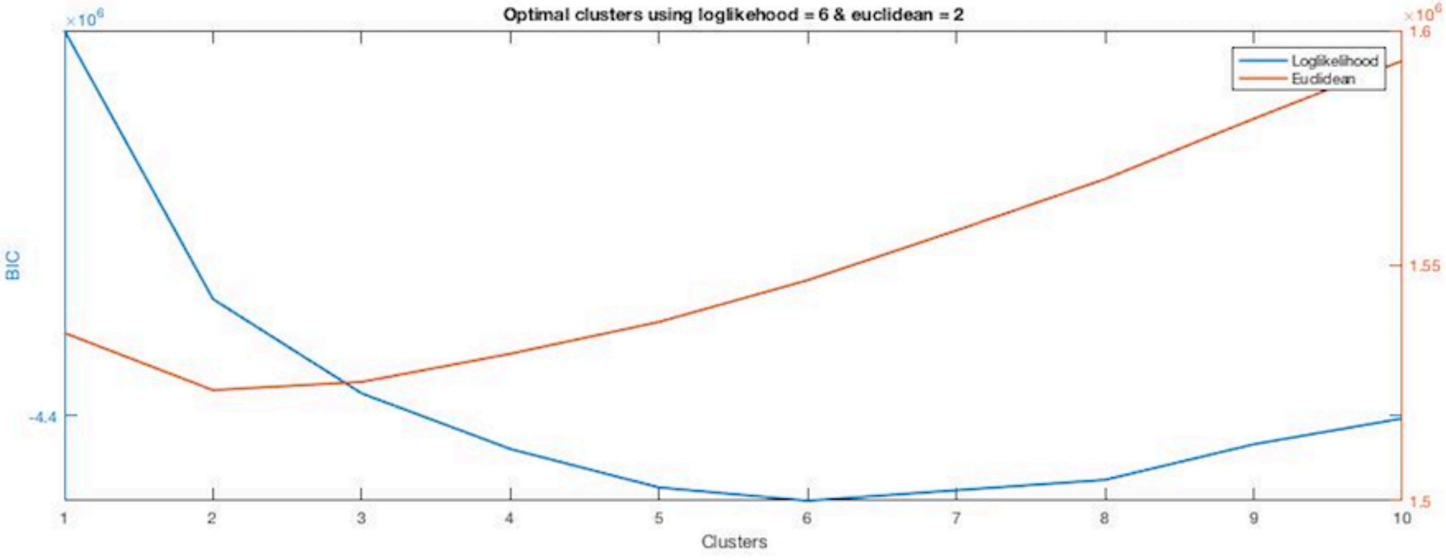

c)

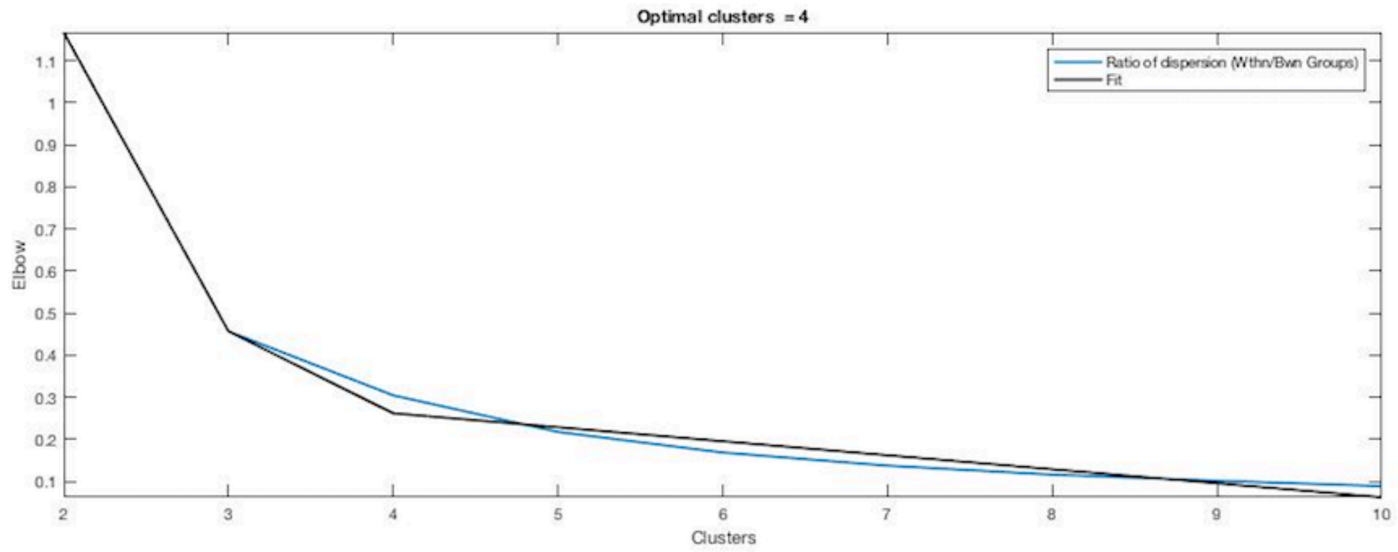

Optimal clusters = 4
Ratio of dispersion (Wthn/Bwn Groups)
Fit
Elbow
Clusters

d)

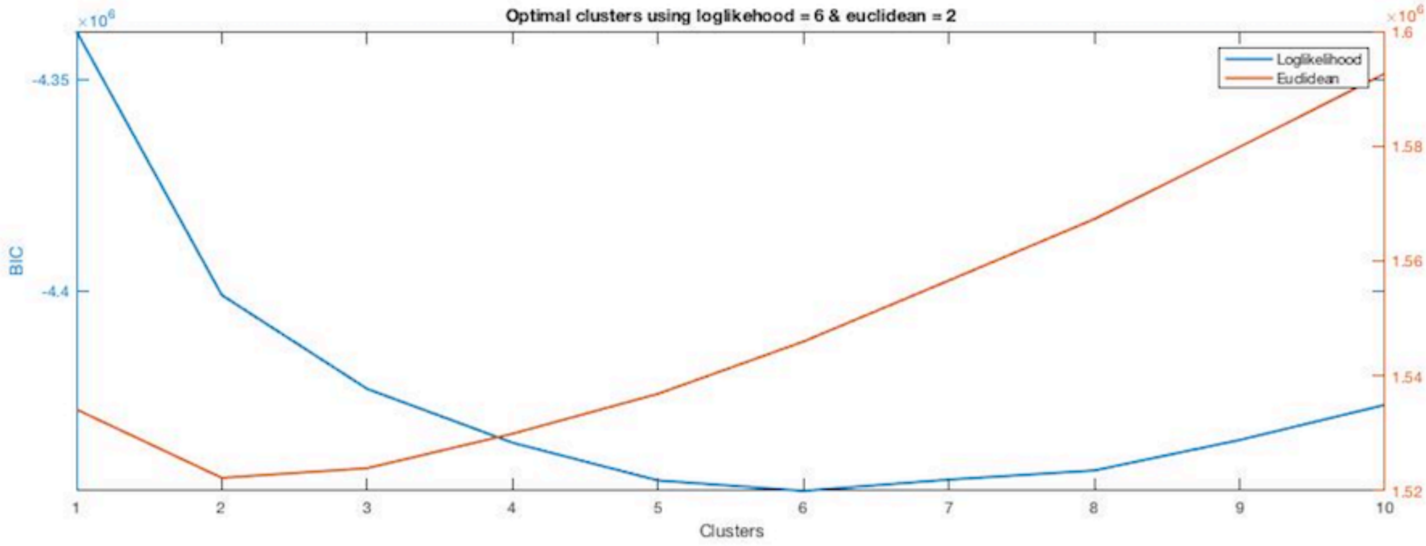

Optimal clusters using loglikehood = 6 & euclidean = 2
Loglikelihood
Euclidean
BIC
Clusters
